# Defective queuosine and i^6^A/ms^2^i^6^A modification of tRNA^Tyr^ cause frameshifting and protein aggregation

**DOI:** 10.1093/nar/gkag664

**Published:** 2026-07-02

**Authors:** Yu Sun, Navpreet Kaur, Hina Zain, Rodrigo Arias-Cartin, Bibek Hamal, Caroline Kühne, Marc Erhardt, Frédéric Barras, Patrick A Limbach, Ann E Ehrenhofer-Murray

**Affiliations:** Institute of Biology, Lebenswissenschaftliche Fakultät, Humboldt-Universität zu Berlin, 10115Berlin, Germany; Institute of Biology, Lebenswissenschaftliche Fakultät, Humboldt-Universität zu Berlin, 10115Berlin, Germany; Department of Chemistry, Rieveschl Laboratories for Mass Spectrometry, University of Cincinnati, Cincinnati, OH 45221, USA; Department of Microbiology, Unit Stress Adaptation and Metabolism in Enterobacteria, Institut Pasteur, Université Paris Cité, UMR CNRS 6047, 75015 Paris, France; Department of Chemistry, Rieveschl Laboratories for Mass Spectrometry, University of Cincinnati, Cincinnati, OH 45221, USA; Institute of Biology, Lebenswissenschaftliche Fakultät, Humboldt-Universität zu Berlin, 10115Berlin, Germany; Institute of Biology, Lebenswissenschaftliche Fakultät, Humboldt-Universität zu Berlin, 10115Berlin, Germany; Department of Microbiology, Unit Stress Adaptation and Metabolism in Enterobacteria, Institut Pasteur, Université Paris Cité, UMR CNRS 6047, 75015 Paris, France; Department of Chemistry, Rieveschl Laboratories for Mass Spectrometry, University of Cincinnati, Cincinnati, OH 45221, USA; Institute of Biology, Lebenswissenschaftliche Fakultät, Humboldt-Universität zu Berlin, 10115Berlin, Germany

## Abstract

Queuosine (Q) modification at the wobble position (Q34) of tRNAs fine-tunes translational speed but is not essential for viability, leaving its physiological role unclear. In bacteria, Q34 is synthesized *de novo*, whereas eukaryotes obtain queuosine (Q) or its precursor queuine (q) from external sources. Q34 uniquely co-occurs with N^6^-isopentenyladenosine (i^6^A) or its derivative 2-methylthio-N^6^-isopentenyladenosine (ms^2^i^6^A) at position 37 of tRNA^Tyr^. We show that loss of Q34 (*∆tgt*) causes a severe growth defect in *Escherichia coli* lacking ms^2^i^6^A due to deletion of the MiaA isopentenyltransferase (*∆miaA*), which is rescued by tRNA^Tyr^ overexpression. Simultaneous absence of Q34 and ms^2^i^6^A37 increases +1 frameshifting at tyrosine codons and promotes protein aggregation, indicating impaired tRNA^Tyr^ function. This functional interplay is evolutionarily conserved: Q34 deficiency aggravates the growth defect of *Schizosaccharomyces pombe* lacking the isopentenyltransferase Tit1 and thus i^6^A. In *S. pombe*, Q34 enhances tRNA^Tyr^ abundance in *tit1∆* cells and reduces i^6^A37 levels in wild-type, revealing reciprocal regulation. Together, these findings demonstrate a synergistic role of Q34 and (ms^2^)i^6^A37 in maintaining translational fidelity and proteostasis, with potential implications for human health when Q availability is limited.

## Graphical Abstract

**Graphical Abstract:**
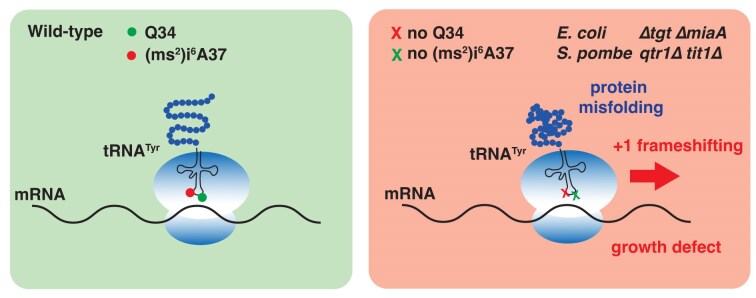

## Introduction

Transfer RNAs (tRNAs) are the most extensively and diversely modified RNAs across all domains of life. Modifications within the anticodon loop fine-tune codon recognition and thereby influence translational speed and fidelity, whereas modifications in the tRNA body support structural stability, folding, localization, and quality control [1]. Deficiencies in tRNA modification are linked to human diseases, including mitochondrial dysfunction, neurodevelopmental disorders, and cancer [2], underscoring their broad biological importance.

**Figure 1.**
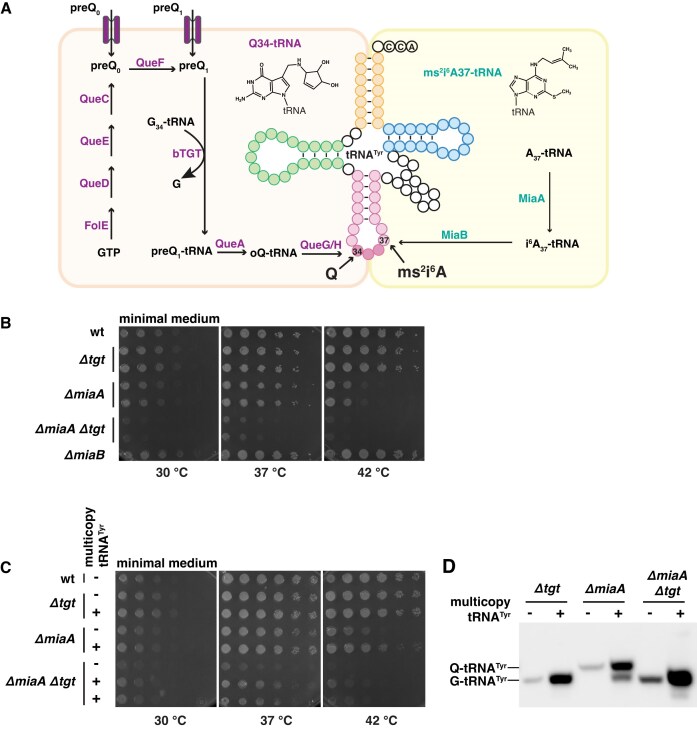
The combined loss of Q34 *(∆tgt*) and ms^2^i^6^A37 (*∆miaA*) causes a growth defect in *E. coli*. (**A**) Schematic of the biosynthetic pathways for queuosine (Q, left) and ms^2^i^6^A modifications (right) on tRNA^Tyr^ in *E. coli*. (**B**) Simultaneous deletion of *tgt* and *miaA* results in a growth defect. The indicated *E. coli* strains were serially diluted 10-fold and spotted on M9 plates supplemented with vitamins and trace elements. Plates were incubated at 30, 37, or 42°C for 18 h. (**C**) The *∆miaA ∆tgt* growth defect is suppressed by overexpression of tRNA^Tyr^. Strains were transformed with either a plasmid expressing tRNA^Tyr^ under the control of the isopropyl β-D-1-thiogalactopyranoside (IPTG)-inducible promoter Ptrc (+) or a vector control (−). Transformants were diluted as in panel (B) and spotted on M9 plates supplemented with vitamins, trace elements, ampicillin, and 0.05 mM IPTG. (**D**) Overexpression of tRNA^Tyr^ in the *E. coli* strains as in panel (C) shows that tRNA^Tyr^ is partially Q-modified in *∆miaA*. APB-Northern blot analysis of tRNA^Tyr^ from the indicated strains grown at 37°C. Q modification causes slower migration in APB gels.

Among the tRNA modifications, queuosine (Q) stands out as one of the most complex modifications and is evolutionarily conserved. Q is a hypermodified 7-deaza-guanosine analog found at the Wobble position (position 34) of tRNAs decoding NAC/U codons (asparagine, aspartate, histidine, and tyrosine codons) [3, 4]. Eubacterial Q biosynthesis begins with five consecutive enzymatic steps using GTP to generate the precursor preQ_1_ [5], which is incorporated into tRNAs by the bacterial tRNA-guanine transglycosylase (bTGT) and is ultimately converted to Q [6–9] (Fig. 1A, left). In contrast, eukaryotes lack *de novo* biosynthesis and instead obtain the Q nucleoside and its nucleobase queuine (q) from diet and/or the gut microbiota. Following hydrolysis of Q to release the q base by Qng1 [10, 11], the eukaryotic TGT (a heterodimer of QTRT1 and QTRT2) incorporates q into tRNAs by replacing G34 [12, 13]. Importantly, Q stimulates 5-methyl-cytosine formation at position 38 (m^5^C38) of tRNA^Asp^ in organisms ranging from *Schizosaccharomyces pombe* and *Dictyostelium discoideum* [14] to *Entamoeba histolytica* [15], mice, and human cells [16, 17].

As a conserved Wobble modification, Q fine-tunes translation in an organism- and codon-specific manner. In *S. pombe*, Q accelerates decoding of C-ending codons in tRNA^Asp^ and tRNA^His^, while slowing U-ending codons in tRNA^Asn^ and tRNA^Tyr^ [17]. In *Trypanosoma brucei*, Q-modified tRNAs preferentially decode U-ending codons [18, 19], a preference also observed in human and mouse cells, although the effects vary by cell type [16, 20]. Q modification is dispensable under normal conditions but becomes important in some organisms during stress. It protects *E. histolytica* from oxidative damage by promoting translation of stress response genes [15], and Q levels rise in mammalian cells exposed to arsenite, which supports the translation of proteins involved in energy metabolism [21]. Q deficiency causes a translational imbalance, which in turn triggers mild protein aggregation [16], and in female mice leads to impaired learning and memory [20, 22]. These findings, together with evidence that queuine availability is regulated by the diet and gut microbes, have attracted growing attention to q as a potential therapeutic agent in neurodegenerative and inflammatory diseases [23].

Despite the evolutionarily conserved role of Q modification in translation, Q deficiency causes no or only mild defects. Here, we explored whether the combined absence of Q and a second tRNA modification caused an enhanced cellular defect. A strong candidate is N^6^-isopentenyladenosine (i^6^A) at position 37 of tRNA^Tyr^_GUA_, which lies near Q34. In eukaryotes, i^6^A37 occurs on both cytosolic (cy-) and mitochondrial (mt-) tRNAs, with species-specific differences in the affected tRNA subsets [24]. In bacteria, i^6^A can undergo further methylthiolation to ms^2^i^6^A, which is also present in mammalian mt-tRNAs [25]. These modifications enhance codon–anticodon interactions, suppress frameshifting, and promote stress adaptation by enabling efficient translation of rare-codon regulators such as RpoS [26, 27]. The absence of i^6^A leads to sensitivity to the target of rapamycin (TOR) inhibitor rapamycin and to mitochondrial dysfunction in *S. pombe* [24, 28], and mutations in the human i^6^A37-modifying enzyme TRIT1 result in a mitochondrial disorder characterized by epilepsy, myoclonic jerks, and psychomotor delay [29, 30].

Previous work showed that bacterial *tgt*, which is required for Q34 formation, and *miaA*, which performs i^6^A37 modification leading to ms^2^i^6^A, are both necessary for full expression of the virulence regulator VirF in *Shigella flexneri* [31]. Absence of either gene reduces VirF protein levels without affecting *virF* mRNA levels. It was proposed that *virF* mRNA itself might be Q-modified, since *Escherichia coli* TGT can Q-modify *virF* mRNA *in vitro* [32]. However, whether *virF* mRNA is Q-modified *in vivo* has not been determined.

In this study, we report a strong genetic interaction between Q34 and ms^2^i^6^A/i^6^A37 of tRNA^Tyr^_GUA_. The combined loss of both Q34 and ms^2^i^6^A37/i^6^A37 caused pronounced growth defects in both *E. coli* and *S. pombe*. The simultaneous absence of these modifications increased frameshifting and enhanced protein aggregation in *E. coli*. Interestingly, in *S. pombe*, the presence of Q modification counteracted i^6^A37 installation on tRNA^Tyr^ in wild-type cells and increased tRNA^Tyr^ expression in the absence of i^6^A37. Altogether, our findings show that Q availability becomes crucial when ms^2^i^6^A or i^6^A are deficient. This suggests that queuine availability may become particularly critical for human health when other tRNA modifications are impaired.

## Materials and methods

### Strains and plasmids

All bacterial and yeast strains used in this study are listed in Supplementary Tables S1–S3. *E. coli* deletion mutants and ibpA–msfGFP fusion were generated by λ-Red recombineering as previously described [33] and verified by direct polymerase chain reaction (PCR) of individual colonies. To construct the *ΔmiaA Δtgt* double mutant, the FRT-flanked kanamycin resistance cassette was excised from the *Δtgt* strain by transient expression of FLP recombinase from the temperature-sensitive plasmid pCP20, and the resulting antibiotic-sensitive *∆tgt* strain was used for subsequent *miaA* deletion. To construct the ibpA–msfGFP strain at *ibpA* locus, a FRT–msfGFP flanked kanamycin resistance cassette was amplified from pRACmsfGFP, and the FRT kanamycin resistance cassette was excised using plasmid pCP20. For protein aggregation assays, *Δtgt, ΔmiaA*, and *ΔmiaA Δtgt* mutants were generated in *E. coli* MG1655 background carrying a chromosomally integrated *ibpA*–msfGFP reporter. The *S. flexneri* mutants (*ΔqueC, Δtgt*, and *ΔqueC Δtgt*) were constructed using λ-Red recombineering following the same strategy and antibiotic resistance marker system as described for *E. coli*. All *S. pombe* strains were derived from AEP1 (FY7385, *h^-^ leu1-32 ura4-D18 his3-D3*). The *tit1* (*SPAC343.15*) and *qtr1* (*SPAC1687.19c*) open reading frames were replaced by KanMX and NatMX resistance cassettes, respectively, by homologous recombination.

Plasmids are listed in Supplementary Table S4, and were generated using standard cloning procedures followed by Sanger sequencing. Oligonucleotides used for strain construction and verification are provided in Supplementary Table S5.

### Cell growth assays

*E. coli* strains were grown overnight in LB medium at 37°C. The overnight cultures were then diluted to an optical density at 600 nm (OD_600_) of 0.1 in 5 ml of fresh medium (LB or M9 minimal medium supplemented with 0.4% glucose as the carbon source) and grown at 37°C to an OD_600_ of approximately 0.5. Suspensions of OD_600_ of 0.5 were generated, and 10-fold serial dilutions were spotted on agar plates.

*S. pombe* strains were grown overnight at 30°C in YES or EMM medium with appropriate supplements. The overnight culture was adjusted to an OD_600_ of 2, and 6-fold dilutions were spotted on agar plates. Where indicated, rapamycin supplementation was at a final concentration of 50 ng/ml, and queuine was supplemented at 100 nM.

### Determination of protein aggregation using IbpA–msfGFP

*E. coli* strains were grown in LB at 37°C to mid-log phase (OD_600_ = 0.4–0.6), washed twice in 1 $\times$ PBS, and finally resuspended in 1 $\times$ PBS. Subsequently, 2 µl of cell suspension was immediately transferred to 1% agarose pads (prepared from Invitrogen Ultrapure Agarose in 1 $\times$PBS), placed on a microscopy slide, and mounted with a cover glass (No. 1.5H coverslips, Marienfeld). A Gene Frame (Thermo Scientific) was used to hold the cover glass on the microscopy slide. GFP fluorescence was imaged using a Nikon Eclipse Ti2 inverted microscope equipped with a 100 $\times$ CFI Plan Apochromat Lambda oil-immersion objective, with 488 nm excitation and 100 ms exposure. Images were processed using identical setting.

Background subtraction was performed in Fiji. Cell segmentation was conducted with Omnipose using the *cyto2* model. Foci were quantified in Fiji using the MicrobeJ plugin with Maxima detection and the Point model. For wild-type, *Δtgt*, and *ΔmiaA* mutants, a tolerance value of 300 was applied, whereas for the double knockout strain, which exhibited smaller and more dispersed fluorescence signals, the tolerance was reduced to 200. Only signals with an intensity ≥300 were considered valid foci. Foci numbers were analyzed using custom R scripts. Statistical significance was assessed by one-way ANOVA followed by Tukey’s HSD test. Four biological replicates were analyzed, with >500 cells quantified per cell.

### Small RNA extraction

Small RNAs were extracted according to the manufacturer’s instructions [PureLink™ miRNA Isolation Kit (Invitrogen)] with some modifications. Cells were lysed in 1 ml of TRIzol (Ambion), followed by the addition of 0.2 ml of chloroform and glass beads. The samples were vortexed for 2 min and centrifuged at 16 000 ×* g* for 15 min at 4°C. The upper phase was mixed with 215 μl of absolute ethanol, applied to a spin cartridge, and centrifuged at 12 000 × *g* for 1 min. The flow-through was combined with 700 μl of absolute ethanol, transferred to a fresh spin cartridge, and centrifuged at 12 000 × *g* for 1 min. After washing with the wash buffer from the kit, small RNAs were eluted in 50 μl of diethyl pyrocarbonate (DEPC)-treated water. Small RNAs were deacylated for 30 min at 37°C in 100 mM Tris–HCl (pH 9.0).

### Acryloyl aminophenylboronic acid (APB) gels and Northern blotting

For the detection of Q modification, 300 ng of deacylated small RNAs were mixed with an equal volume of 2 $\times$ RNA loading buffer, denatured at 70°C for 5 min, and resolved on a 12% urea–TBE polyacrylamide gel (7 M urea) containing 5 mg/ml 3-(acrylamido)-phenylboronic acid. Electrophoresis was carried out in 1 $\times$ TBE buffer at 30 mA for approximately 2 h. Following electrophoresis, RNAs were blotted to a Biodyne B Nylon membrane (0.45 μm) in 0.5 $\times$ TBE buffer at 4°C, 150 mA for 90 min. The membrane was first blocked with DIG Easy Hyb buffer (Roche), followed by hybridization overnight at 60°C with a denatured biotinylated probe. The signal was detected using the Chemiluminescent Nucleic Acid Detection Module Kit (Thermo Fisher Scientific). Imaging was carried out using a ChemiDoc Imaging System (Bio-Rad). Probes used in this study are provided in Supplementary Table S6.

### Analysis of ms^2^i^6^A by high-throughput sequencing

#### Sequencing library preparation

ms^2^i^6^A was determined by measuring error incorporation during reverse transcription using the RT-KTq I614Y polymerase. For this purpose, small RNAs were extracted from *E. coli* strains grown in M9 medium at either 37°C or 42°C. Library preparation was carried out based on our previously published protocol [34], with modifications tailored for ms^2^i^6^A modification detection. In brief, 500 ng of deacylated small RNAs were hybridized with 1 μl of 10 μM tRNA^Tyr^ stem-loop primer (Supplementary Table S7) using the following thermocycling program: 95°C for 2 min, 65°C for 30 s, followed by a gradual ramp to 4°C at 5°C per 30 s. After incubation on ice for 1 min, reverse transcription was performed in 20 μl reactions containing 100 μM dA/C/GTP, 12.5 μM dTTP, 100 nM RT-KTq I614Y enzyme, and RT buffer [50 mM Tris–HCl (pH 9.2), 16 mM (NH_4_)_2_SO_4_, 2.5 mM MgCl_2_, and 0.1% Tween-20], and incubation at 55°C for 1 h.

Following reverse transcription, 5 μl of complementary DNA (cDNA) was used for PCR amplification in a 50 μl of reaction containing 0.5 µM forward and reverse primers, 250 μM dNTPs, 2 mM MgCl_2_, 1 $\times$ Taq buffer with KCl, and 1.25 U Taq polymerase (Thermo Fisher). The thermal cycling conditions were 96°C for 3 min, 40 cycles of 96°C for 30 s, 50°C for 45 s, and 72°C for 45 s, followed by a final extension at 72°C for 5 min. 3′-End primers included sample-specific barcodes for multiplexing. PCR products were resolved on a 2% agarose gel and purified using the QIAquick Gel Extraction Kit (Qiagen). The purified PCR products were pooled and submitted for next-generation sequencing (Eurofins). All experiments were performed in three biological replicates.

#### Calibration samples for relative quantification of ms^2^i^6^A

Calibration samples for the relative quantification of ms^2^i^6^A were prepared by mixing small RNAs isolated from the *∆miaA* strain (0% ms^2^i^6^A) with small RNAs from the wt strain (ms^2^i^6^A-containing, absolute modification level unknown) to a final amount of 500 ng. The resulting mixtures contained 0%, 12.5%, 25%, 50%, 75%, and 100% wt-type small RNAs. Sequencing libraries from these calibration samples were prepared using the same protocol as described above. All calibrations were performed in three biological replicates.

#### Data analysis for ms^2^i^6^A detection

For each run, at least 10 million sequencing reads were generated from NGSelect amplicon sequencing. The read length was 151 nt, which is sufficient to cover the entire tRNA sequence. Sequencing reads were initially demultiplexed based on barcode sequences, and high-quality reads were selected using custom R scripts. For alignment, a reference sequence was constructed comprising two *E. coli* tRNA^Tyr^ isodecoders (from Ensembl Bacteria) along with the 3′ and 5′ primer regions. The 3′ and 5′ primer regions were retained during alignment, but barcode positions were substituted with “NNNN” to prevent mismatches across different barcodes from affecting the alignment. Read alignment was carried out using Bowtie2 with parameters -N 1 -L 6 to allow up to one mismatch per 6 nt seed. To assess reverse transcription-derived misincorporation patterns, the misincorporation rate at each nucleotide position was quantified using CoverageAnalyzer [35] on aligned SAM files.

### Analysis of i^6^A by high-throughput sequencing

#### Sequencing library preparation for i^6^A detection

Small RNAs for i^6^A detection were isolated from *S. pombe* strains cultured in YES medium at 30°C, with or without queuosine (final concentration 100 nM). Iodine treatment for the cyclization of i^6^A was carried out according to the IMCRT tRNA-seq method [36]. In brief, deacylated small RNAs were further treated with 0.5 M I_2_ for 20 min on ice. Following iodine treatment, saturated Na_2_S_2_O_3_ was added to the reaction mixture until the solution became colorless. Subsequently, saturated Na_2_CO_3_ was added dropwise until effervescence ceased. Upon completion of the reaction, small RNAs were purified using the RNA Clean & Concentrator Kit (ZYMO Research) according to the manufacturer’s instructions. Iodine-treated samples were then used for sequencing library preparation following the same protocol as described above for ms^2^i^6^A detection. Primers used for i^6^A are listed in Supplementary Table S8.

#### Calibration samples for relative quantification of i^6^A

Calibration samples for the relative quantification of i^6^A were generated by mixing iodine-treated small RNA from the *tit1Δ* strain (grown in YES medium; 0% i^6^A) with iodine-treated small RNA from the wt strain (grown in YES medium; i^6^A-containing, absolute modification level unknown), to a final total of 500 ng RNA per sample. The resulting mixtures were prepared to contain 0%, 30%, 40%, 50%, 60%, 70%, 80%, 85%, 90%, 95%, and 100% wt-type small RNAs. The calibration samples were subsequently used for sequencing library preparation. All calibrations were performed in three biological replicates.

#### Data analysis for i^6^A detection

Data processing for i^6^A detection followed the same pipeline used for ms^2^i^6^A detection in *E. coli* samples using a customized reference sequence composed of *S. pombe* mature tRNA^Tyr^ isodecoders and intron-containing tRNA^Tyr^ isodecoders (PomBase).

#### Detection of preQ_1_-L1 modification in RNAs of *S. flexneri*

To assess Q modification in *S. flexneri virF* mRNA, cells were metabolically labeled with the azide-functionalized preQ_1_ derivative (preQ_1_-L1) [37], followed by CuAAC-mediated fluorescent or biotin labeling.

#### Total RNA extraction

*S. flexneri* strains were grown overnight at 37°C in M9 minimal medium supplemented with vitamins and trace elements, diluted to an OD_600_ of 0.1 in fresh M9 minimal medium supplemented with vitamins, trace elements and 10 μM preQ_1_-L1, and grown at 37°C to mid-log phase. Total RNA was extracted by adding 1 ml of phenol and glass beads. Cells were lysed by vigorous shaking for 5 min, followed by centrifugation at 20 000 ×* g* for 5 min. The aqueous phase was then extracted by adding an equal volume of phenol/chloroform/isoamyl alcohol and centrifuged at 20 000 ×* g* for 5 min. The upper phase was further purified by mixing with an equal volume of chloroform, followed by centrifugation at 20 000 ×* g* for 5 min. RNAs were precipitated at −80°C for at least 1 h with 0.7 volume of isopropanol. The RNA pellet was washed with 70% ethanol, air dried, and resuspended in DEPC-treated water.

### CuAAC click reaction

10 μg of total RNA was incubated for 2 h at 25°C in the dark in 50% dimethyl sulfoxide (DMSO) containing 5 mM tris(3-hydroxypropyltriazolylmethyl)amine (THPTA), 5 mM sodium ascorbate, 0.5 mM CuSO_4_, and 50 μM AlexaFluor 594 alkyne or biotin-PEG4-alkyne (Thermo Fisher Scientific). Labeled RNA was ethanol-precipitated, washed twice with 70% ethanol, and resuspended in DEPC-treated water.

### Detection of preQ_1_-L1 incorporation by denaturing PAGE

RNA labeled with AlexaFluor 594 alkyne was analyzed by denaturing polyacrylamide gel electrophoresis. Samples were separated on a 10% polyacrylamide gel containing 8 M urea in 1 $\times$ TBE buffer. Fluorescently labeled RNA was visualized using the Typhoon 9500 (GE Healthcare) with 532 nm laser excitation. To visualize total RNA, the gel was subsequently stained with SYBR gold nucleic acid stain (Thermo Fisher Scientific) for 10 min and imaged using the Typhoon scanner.

### Detection of preQ_1_-L1 incorporation by RNA immunoprecipitation combined with RT-qPCR

Beads were washed and equilibrated in B&W buffer, incubated with 3 µg of biotin-labeled RNA at room temperature for 1 h, washed twice with 1 $\times$ B&W buffer, and bound RNA was eluted in nuclease-free water at 95°C for 10 min. Input (pre-purification) and IP (post-purification) RNA were subjected to RT-qPCR. cDNA was synthesized from 300 ng RNA using SuperScript III and stem-loop primers listed in Supplementary Table S9. qPCR was performed using SYBR Green Master Mix with 95°C for 2 min, 40 cycles of 95°C for 10 s, 58°C for 15 s, and 72°C for 20 s, followed by melting curve analysis.

### Detection of Q modification in *virF* mRNA by RT-KTq I614Y

Q modification in *virF* mRNA was analyzed by RT-KTq I614Y-mediated amplicon sequencing as described above, using 500 ng of total RNA and stem-loop primers listed in Supplementary Table S9. Reverse transcription was performed with 100 µM each dNTP, or with reduced dCTP concentrations of 25, 12.5, or 6.25 µM where indicated. Primers annealing to the 3′-end contained barcoded sequences for multiplexing of PCR products. Libraries for HTS were generated as above, and analysis of the resulting products was performed as described above for ms^2^i^6^A and i^6^A detection.

### tRNA nucleoside analysis

For nucleoside analysis by LC-MS/MS, 4 µg of total tRNAs were digested in a mixture containing 24.8 mM NH_4_OAc (pH 5), 22 μM ZnCl_2_, 0.3 U nuclease P1, and 0.1 U snake venom phosphodiesterase. The mixture was incubated at 37°C for 2 h. FastAP thermosensitive alkaline phosphatase (1 U) and FastAP reaction buffer were then added with additional incubation at 37°C for 1 h. After incubation, the digested samples were dried with a SpeedVac concentrator (Thermo Fischer Scientific). Samples were analyzed on a TSQ Quantiva Triple Quadrupole mass spectrometer (Thermo Fischer Scientific) in positive ion mode. Nucleosides were separated by reversed phase-liquid chromatography using a HSS T3 column (1 $\times$ 100 mm, 1.8 μm particle; Waters Corporation, Milford, MA, USA). The column was operated at 30°C with a flow rate of 0.1 ml/min using 5.3 mM NH_4_OAc in water (pH 4.5) as mobile phase A (MPA) and 40% ACN with 5.3 mM NH_4_OAc as mobile phase B (MPB). The gradient of MPB was as follows: 0% from 0 to 7.6 min, 2% at 15.7 min, 3% at 19.2 min, 5% at 25.7 min, 25% at 29.5 min, 50% at 32.3 min, 75% at 36.4 min to 36.6 min, 99% from 39.6 min to 46.8 min, and back to 0% at 46.9 min. Sheath gas, auxiliary gas, and sweep gas at the ionization source were set to 30.0, 5.0, and 0.0 (au), respectively. The spray voltage was 3500 V, ion transfer tube temperature was 290.0°C, and vaporizer temperature was 150°C. Data were acquired via Selected Reaction Monitoring (SRM) mode using the parameters in Supplementary Table S10. Three technical replicates were analyzed for each sample. Nucleoside identification was based on the molecular ion (MH+), nucleobase product ion (BH2+), and appropriate chromatographic retention time. Data analysis and interpretation was performed using Xcalibur software (Thermo Fisher Scientific).

### tRNA oligonucleotide analysis

For oligonucleotide analysis by LC-MS/MS, 4 µg of isolated total tRNAs were suspended in water and denatured at 95°C for 2 min and then immediately cooled at 4°C. RNase T1 (50 U/µg) (Worthington Biochemical) was used for digestion in 110 mM ammonium acetate buffer. The samples were incubated at 37°C for 2 h, dried in a SpeedVac concentrator (Thermo Fischer Scientific), and dissolved in mobile phase A for analysis. LC-MS/MS analyses were performed using an Ultimate 3000 Ultra-High-Performance Liquid Chromatography (UHPLC, Thermo Scientific) coupled with a Waters Synapt G2-S (Quadrupole time-of-flight, Q-TOF) mass spectrometer operating in negative ion mode. Oligonucleotides are separated by ion-pair reversed phase chromatography using mobile phase A [8 mM triethylamine (TEA) and 200 nM hexafluoroisopropanol (HFIP), pH 7.8 in water] and mobile phase B [8 mM TEA and 200 mM HFIP, pH 7.8 in methanol] at 60°C. The column used for separation was a nanoEase peptide BEH C18 column (300 µm $\times$ 150 mm, 1.7 µm particle size, 130 Å, Waters Corporation). The gradient includes the initial hold for 2 min at 3% B for sample to load, followed by a ramp to 55% B at 70 min, ramping to 99% with a 5 min hold at 99% B and re-equilibration at 3% B for 30 min. The flow rate was 5 µl min^−1^. The ESI parameters were 2.5 kV source voltage, 30 V sample cone, source and desolvation temperatures were 120°C and 400°C, respectively, and cone and desolvation gas flow rates were 50 and 800 L h^−1^, respectively. A scan range of 545–2000 *m/z* (0.5 s) was employed for first-stage (MS) data acquisition and 250–2000 *m/z* (1 s) for second (MS/MS)-stage data acquisition. The top three most abundant ions in the first stage were chosen to face fragmentation for MS/MS using an *m/z*-dependent collision energy profile (20–23 V at *m/z* 545; 51–57 V at *m/z* 2000) before exclusion for 60 s using the dynamic exclusion feature. Mongo Oligo mass calculator (https://mstoolbox.github.io/mongo/) was used to predict the *m/z* values for RNase T1 digestion products and expected MS/MS fragmentation patterns. Data were interpreted and analyzed using Masslynx software (Waters).

## Results

### Absence of both Q34 and ms^2^i^6^A37 modification causes a pronounced growth defect in *E. coli*

In *E. coli*, bTGT incorporates preQ_1_ into Q-tRNAs, which are further modified to mature Q. Among the four Q-tRNAs, tRNA^Tyr^ is modified at the nearby position 37 to i^6^A by MiaA and then to ms^2^i^6^A37 by MiaB (Fig. 1A, right; see Supplementary Fig. S1 for co-occurrence of Q34 and ms^2^i^6^A37 on *E. coli* tRNA^Tyr^ by LC-MS/MS oligonucleotides analysis). To evaluate a possible interplay between Q34 and ms^2^i^6^A37, we investigated whether the absence of both Q and ms^2^i^6^A leads to a growth defect in *E. coli*. For this purpose, growth assays were performed using *E. coli* strains that were wild-type (wt), lacked either Q34 or ms^2^i^6^A37 modification (*∆tgt* or *∆miaA*), or strains lacking both Q34 and ms^2^i^6^A37 modification (*∆miaA ∆tgt*). As expected, *∆miaA*, but not wt or *∆tgt*, showed a growth defect at elevated temperatures (Fig. 1B) [38]. Strikingly, deletion of both *tgt* and *miaA* caused a strong temperature-sensitive growth defect on minimal medium (Fig. 1B), thus showing a genetic interaction between *tgt* and *miaA*. A mild growth defect of *∆miaA ∆tgt* was also observed on rich medium (Supplementary Fig. S2A). Furthermore, expression of *tgt* or *miaA* in *trans* complemented the growth defect of the *∆miaA ∆tgt* double mutant, and *tgt* restored Q modification of tRNA^Tyr^ (Supplementary Fig. S2B–D). Of note, *miaA* expression enhanced, rather than decreased, the growth defect of *∆miaA* alone, as has been reported earlier [38]. Taken together, these results showed that the combined absence of *tgt* and *miaA*, and thus Q34 and ms^2^i^6^A37 modification on tRNAs, strongly compromises *E. coli* growth.

### Growth defect of *∆miaA ∆tgt* is due to deficient tRNA^Tyr^ modification

In some instances, the growth defects observed in mutants lacking tRNA-modifying genes can be rescued by overexpression of the corresponding tRNAs [39]. Since tRNA^Tyr^ is the only common substrate of MiaA and bTGT, we next examined whether its overexpression could rescue the growth defect of *∆miaA ∆tgt*. Importantly, tRNA^Tyr^ overexpression partially suppressed the growth defect of *∆miaA ∆tgt* and *∆miaA* (Fig. 1C). Plasmid-based tRNA^Tyr^ was overexpressed ~10-fold, as determined by Northern blotting of a gel containing acryloylaminophenyl boronic acid (APB), which causes slower migration of Q-modified tRNAs [40]. Of note, overexpressed tRNA^Tyr^ in *∆miaA* was not fully Q-modified, indicating a limiting capacity of the Q modification pathway in this context (Fig. 1D; exposure was adjusted to show the level of tRNA^Tyr^ overexpression, c.f. Supplementary Fig. S2D). However, the aminoacylation level of tRNA^Tyr^ was unaffected by overexpression or Q/ms^2^i^6^A modification (Supplementary Fig. S2E). Taken together, this showed that the growth defect of *∆miaA ∆tgt* stems primarily from impaired tRNA^Tyr^ function due to the combined loss of both Q34 and ms^2^i^6^A37 modifications in the anticodon loop.

### *∆miaA ∆tgt* disrupts translational fidelity and enhances protein aggregation in *E. coli*

The bulky ms^2^i^6^A modification at position 37 lies immediately 3′ to the GUA anticodon in tRNA^Tyr^, and it compensates for the weaker A-U base-pairing at position 36, thus stabilizing the codon–anticodon interaction and preventing ribosome slippage and frameshifting during translation [41]. The absence of Q34 (*∆tgt*) mildly increases frameshifting at the U-ending codons for tyrosine (Tyr) and histidine (His) in some sequence contexts [42]. Given the growth defect of *E. coli* in the absence of Q34 and ms^2^i^6^A37, it was of interest to investigate whether their combined absence enhanced frameshifting by tRNA^Tyr^.

**Figure 2.**
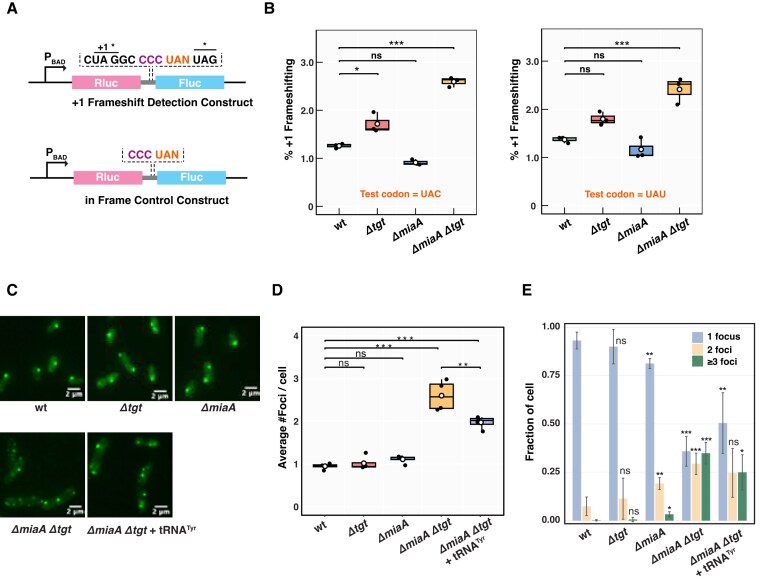
*∆miaA ∆tgt* causes enhanced frameshifting at tyrosine codons and protein aggregation in *E. coli*. (**A**) Schematic of the +1 frameshifting assay using a dual-luciferase reporter. The test codon (UAN) is either the UAU or the UAC tyrosine codon. The in-frame control construct, which lacks a frameshifting window, was used for normalization. R-luc, Renilla luciferase; F-luc: Firefly luciferase. The constructs were expressed under the control of the arabinose-inducible promoter P_BAD_. (**B**) Frameshifting ratios of wt and deletion strains. Individual data points (black dots) represent measurements from three independent transformants, each analyzed in duplicate biological replicates. Open circles indicate group means. Statistical significance was determined by ANOVA with Sidak post hoc tests (ns: *P* > 0.05, *: *P* ≤ 0.05, ***: *P* ≤ 0.001). (**C**) Representative fluorescence images of *E. coli* strains carrying the IbpA–msfGFP reporter, a sensor of misfolded and aggregated proteins. The IbpA–msfGFP reporter is expressed from its native promoter at the endogenous chromosomal locus. Strains were grown in LB medium and suspended in phosphate-buffered saline (PBS); scale bars: 2 μm. (**D**) Quantification of the average number of IbpA–msfGFP foci per cell. Each black dot represents an independent biological replicate (500 cells per data point, *n* = 4). Open circles indicate group means. Statistical significance as in panel (B) (ns: *P* > 0.05, **: *P* ≤ 0.01, ***: *P* ≤ 0.001). (**E**) Distribution of foci counts per cell (same data as in D). Bars represent the mean fraction of cells containing 1, 2, or 3 foci per cell, and error bars indicate the standard deviation (*n* = 4). Statistical comparisons for each foci category were performed against the corresponding wt category. Statistical significance: ns, *P* > 0.05; *, *P* ≤ 0.05; **, *P* ≤ 0.01; ***, *P* ≤ 0.001).

For this purpose, we adopted a dual-luciferase reporter assay, a gene fusion of *Renilla* luciferase (R-Luc) with Firefly luciferase (F-Luc), for expression in *E. coli* [18]. The translation of the downstream F-Luc depends on a programmed +1 frameshift between the two genes (Fig. 2A). Frameshifting efficiency was quantified as the normalized F-Luc/R-Luc luminescence ratio of the +1 frameshift construct relative to an in-frame control. Reporters carrying either UAU or UAC Tyr test codons at the shift site were introduced into wt, *∆tgt, ∆miaA*, and *∆miaA ∆tgt* strains. Interestingly, *∆tgt* (absence of Q34) increased frameshifting by 36.5% at the UAC codon (*P* = 0.0129) and by 31.4% at the UAU codons (*P* = 0.1514) compared to wt, though statistical significance was only observed for the UAC codon (Fig. 2B). Furthermore, *∆miaA* (absence of ms^2^i^6^A) showed a mild reduction in frameshifting in this assay that was not statistically significant. Importantly, *∆miaA ∆tgt* caused a pronounced increase in frameshifting of 105.6% at the C-ending Tyr codon (*P* < 0.0001) and 75.9% at the U-ending Tyr codon (*P* = 0.0010). This shows that the simultaneous absence of Q34 and ms^2^i^6^A37 in tRNA^Tyr^ synergistically enhances frameshifting at Tyr codons.

Based on the elevated frameshifting observed in *∆miaA ∆tgt*, we next asked whether the reduced translational fidelity leads to protein aggregation. To this end, we employed a chromosomal fluorescent reporter of IbpA (IbpA–msfGFP under the endogenous promoter) as a marker for misfolded and aggregated polypeptides [43]. Foci of IbpA protein aggregates were observed in all strains upon nutrient deprivation (Fig. 2C). However, there was a strikingly different aggregation pattern in *∆miaA ∆tgt* compared to the single mutants and the wt strain (Fig. 2C). While wt, *∆tgt*, and *∆miaA* strains typically exhibited a single large focus per cell, *∆miaA ∆tgt* frequently contained multiple smaller foci. Quantitative analysis revealed an average of about 2.60 ± 0.36 foci per cell in the *∆miaA ∆tgt* strain, compared to approximately one in the other strains (Fig. 2D). Furthermore, distribution analysis revealed that wt and *∆tgt* cells were predominantly characterized by single-focus patterns (wt: 93 ± 4%; *∆tgt: 90* ± 9%), whereas *∆miaA* cells exhibited a modest shift toward two foci (19 ± 3%). Notably, *∆miaA ∆tgt* exhibited a profoundly altered distribution, with approximately 36 ± 8% of cells harboring one focus, 29 ± 6% harboring two, and 35 ± 6% harboring three or more foci (Fig. 2E). In the same assay, overexpression of tRNA^Tyr^ in *∆miaA ∆tgt* caused a partial suppression of the protein aggregation defect, with more cells (50 ± 16%) containing a single focus. Quantification revealed that the number of foci per cell (1.98 ± 0.15) was significantly reduced compared to the *∆miaA ∆tgt* strain without tRNA^Tyr^ overexpression, yet remained higher than in wt or either single mutant (Fig. 2E). Of note, IbpA was decreased rather than increased in *∆tgt ∆miaA* (Supplementary Fig. S2F), showing that the increased number of foci was not a result of increased IbpA expression. Taken together, the increased protein aggregation in the *∆miaA ∆tgt* double mutant, which lacks Q and ms^2^i^6^A modifications of tRNA^Tyr^, indicates that the absence of these tRNA modifications in tRNA^Tyr^ causes impaired maintenance of proteome homeostasis due to a defect in translation.

### *virF* mRNA is not Q-modified by bTGT in *Shigella flexneri*

The genetic interaction observed above between *∆tgt* and *∆miaA* suggested that Q34 and ms^2^i^6^A jointly support translation fidelity. This was particularly interesting in light of the observation that both genes are required for efficient translation of the VirF virulence protein in *S. flexneri*. One explanation is that bTGT directly modifies *virF* mRNA *in vivo*. Indeed, bTGT as previously been shown to Q-modify *virF in vitro* [32]. We, therefore, evaluated its modification status *in vivo*. Using a clickable preQ_1_ derivative [37], we confirmed modification of *virF* by bTGT *in vitro* (Supplementary Fig. S3A). Providing the preQ_1_ derivative to *S. flexneri* readily showed *in vivo* incorporation into tRNAs, but no incorporation into *virF* was detected (Supplementary Fig. S3B and C). Similarly, the detection of Q modification by error incorporation upon reverse transcription [34] revealed Q modification of *virF* by bTGT *in vitro*, but not *in vivo* (Supplementary Fig. S3C and D), showing that under physiological conditions, *virF* mRNA is unlikely to be a functionally relevant bTGT target. Thus, the reported dependence of VirF translation on TGT and MiaA is more consistent with an effect through tRNA-dependent translation than an effect via Q modification of *virF* mRNA.

### Combined loss of Q34 and i^6^A37 in *S. pombe* causes sensitivity to rapamycin

**Figure 3.**
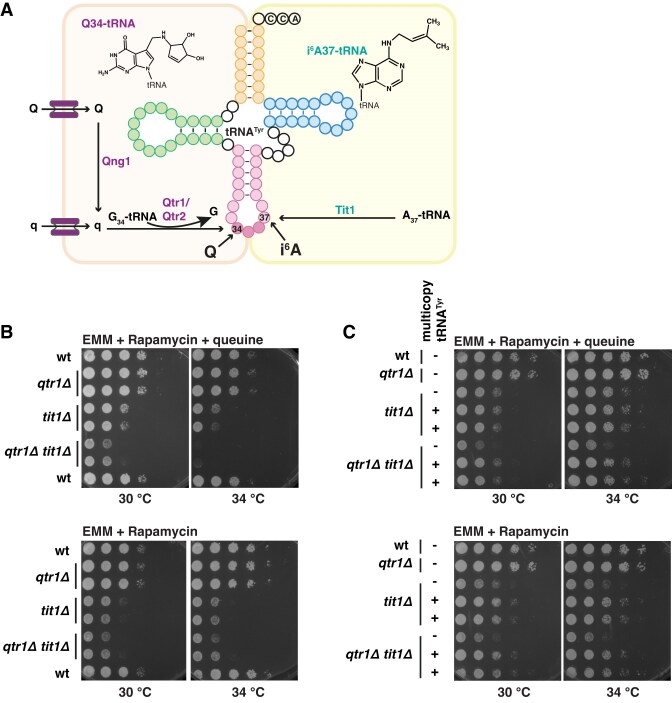
Absence of Q34 modification exacerbates the growth defect of *S. pombe tit1∆* cells, which lack i^6^A37. (**A**) Schematic overview of the biosynthetic pathways for Q (left) and i^6^A modifications (right) on tRNA^Tyr^ in *S. pombe. S. pombe* lacks de novo Q biosynthesis pathway and instead salvages Q and q from the environment. (**B**) Abrogation of Q modification in *tit1∆* causes a strong rapamycin-sensitive growth defect. Serial dilutions of *S. pombe* strains were spotted on minimal medium (EMM) containing rapamycin (50 ng/ml), either with (top) or without (bottom) queuine supplementation (100 nM), and plates were incubated at 30°C or 34°C for 4 days. (**C**) Overexpression of tRNA^Tyr^ partially rescues the rapamycin sensitivity of *qtr1Δ tit1Δ* and *tit1∆*. Strains were transformed with tRNA^Tyr^ plasmid (+) or an empty vector (−). Spot assays were performed as described in panel (B).

We next asked whether the genetic interaction between Q34 and i^6^A37 is conserved in eukaryotes by investigating the effect of their absence in the fission yeast *S. pombe*. Unlike bacteria, *S. pombe* depends on exogenous queuine supplementation, which is incorporated into the Q-tRNAs at position 34 by the heterodimeric eTGT complex (Qtr1/Qtr2 in *S. pombe*) [44, 45]. The i^6^A37 modification is introduced by the Tit1 isopentenyl transferase (Fig. 3A; *S. pombe* has no ms^2^i^6^A37 on tRNA^Tyr^, as confirmed by LC-MS/MS, Supplementary Fig. S4). Growth assays were performed with wt, *qtr1Δ, tit1Δ*, and the *qtr1Δ tit1Δ* double mutant in the presence or absence of rapamycin, with or without q supplementation. Rapamycin inhibits the mTOR signaling cascade to reduce global protein synthesis, but its effect in *S. pombe* is attenuated because TORC1 activity is only partially suppressed [46]. However, loss of Tit1, which eliminates i^6^A37, increases rapamycin sensitivity in *S. pombe* by impairing translational fidelity [28]. Thus, rapamycin was used as translation-related stress condition to test whether defects caused by loss of tRNA modifications are exacerbated when translational homeostasis is challenged. In the presence of q, *qtr1Δ* showed no growth defect, whereas *tit1Δ* displayed increased sensitivity to rapamycin, as expected [28] (Fig. 3B). Importantly, the *qtr1Δ tit1Δ* double mutant exhibited a pronounced growth defect across all temperatures. In contrast, without q, *tit1Δ*, and *qtr1Δ tit1Δ*, which lack both i^6^A37 and Q34, were equally hypersensitive to rapamycin (Fig. 3B). This shows that Q modification ameliorates the growth defect of *tit1∆*. In the absence of rapamycin, *tit1∆* showed mild temperature sensitivity that was marginally suppressed by q supplementation, showing that the growth defect was enhanced by rapamycin (Supplementary Fig. S5).

We further tested whether defective tRNA^Tyr^ was responsible for the rapamycin sensitivity of *qtr1Δ tit1Δ*, as was the case in *E. coli*. Indeed, overexpression of tRNA^Tyr^ partially suppressed the growth defect of *qtr1Δ tit1Δ* (Fig. 3C). The modest suppression may be explained by the observation that overexpression of tRNA^Tyr^ was ~2-fold (Supplementary Fig. S6A). Notably, the endogenous level of tRNA^Tyr^ was not significantly reduced in *qtr1Δ tit1Δ* cells (Supplementary Fig. S6B). Furthermore, the level of aminoacylation was unaffected by overexpression or the absence of Q and i^6^A (Supplementary Fig. S6C). Taken together, these results showed that the loss of Q34 exacerbates rapamycin sensitivity in *S. pombe* cells lacking i^6^A37, and the defect is compensated by increasing the levels of tRNA^Tyr^, the only tRNA carrying both modifications. This highlights a conserved genetic interaction between Q34 and ms^2^i^6^A/i^6^A37 that extends beyond *E. coli* to eukaryotes.

### Queuosine modification causes overexpression of tRNA^Tyr^ in *S. pombe* cells lacking i^6^A

Given the cellular defect in the absence of both Q34 and i^6^A37 in *S. pombe*, it was of interest to investigate the level of both modifications in tRNA^Tyr^. Also, previous studies have demonstrated that the Q34 modification stimulates the formation of m^5^C38 in tRNA^Asp^ by the DNMT2 methyltransferase in eukaryotes [14, 16, 17, 47]. Interestingly, Q34 and ms^2^i^6^A37 occupy relative positions within the anticodon loop of tRNA^Tyr^ that are similar to those of Q34 and m^5^C38 in tRNA^Asp^. This structural resemblance prompted us to investigate whether Q34 and i^6^A37 might similarly engage in a modification circuit, in that the presence or absence of one modification could influence the formation of the other modification.

**Figure 4.**
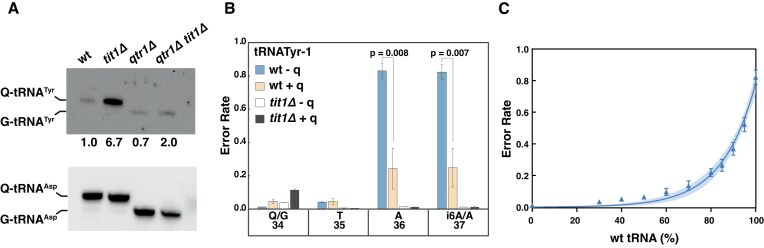
Queuosine modification causes overexpression of tRNA^Tyr^ in *S. pombe*, and Q34 inhibits i^6^A modification in tRNA^Tyr^. (**A**) APB Northern blot analysis reveals Q-dependent overexpression of tRNA^Tyr^ in *tit1*∆. Strains were cultured with q at 30°C, and small RNAs were analyzed by APB Northern blot using a probe for tRNA^Tyr^. Detection of tRNA^Asp^ was used as a loading control. Relative tRNA^Tyr^ levels were normalized to tRNA^Asp^ levels. Values below each lane represent the expression level of tRNA^Tyr^ relative to that of wt (set as 1). Biological replicates are shown in Supplementary Fig. S6D. (**B**) Q modification reduces the i^6^A37 level in tRNA^Tyr^ by approximately 17.5%. Misincorporation profiles of tRNA^Tyr^ were generated from iodine-treated small RNA samples using RT-KTq I614Y. The respective strains were cultured in rich medium with (+q) or without (−q) queuine (*n* = 3). (**C**) Calibration samples, generated by mixing RNA from wt-q and *tit1Δ*-q samples at defined ratios (*x*-axis, fraction of wt), were used to establish a quantitative relationship between misincorporation rate at position 37 and i^6^A levels relative to wt-q. The curve represents an exponential fit (*R*^2^ = 0.9825), with shaded areas indicating the 95% confidence interval.

To determine Q modification levels, APB Northern blotting for tRNA^Tyr^ was performed in wt, *tit1∆, qtr1∆* and *qtr1∆ tit1∆* after treatment with q. This showed that tRNA^Tyr^ was fully Q-modified in both wt and *tit1Δ*, indicating that Q34 incorporation on tRNA^Tyr^ occurs independently of i^6^A37 (Fig. 4A and Supplementary Fig. S6D). Unexpectedly, however, we observed a marked increase of tRNA^Tyr^ levels in *tit1Δ* cells. Normalization to tRNA^Asp^, a Q-modified tRNA that does not carry i^6^A37, revealed that the tRNA^Tyr^ levels in *tit1Δ* were ∼6.7-fold higher than in wt, and the increased level was abrogated by *qtr1∆* and thus loss of Q34 modification. This observation was surprising and suggested that yeast cells compensate for the absence of i^6^A37 in Q-modified tRNA^Tyr^ by increasing its abundance.

### Queuosine reduces i^6^A37 modification of tRNA^Tyr^ in *S. pombe*

To examine whether Q34 influences i^6^A37 modification on tRNA^Tyr^, we adapted an iodine-mediated cyclization and reverse transcription tRNA-seq method by employing the error-prone RT-KTq I614Y polymerase [34] in place of Induro polymerase used in the earlier study [36]. Application of this method revealed high misincorporation rates at nucleotides A36 and i^6^A37 in wt, whereas *tit1Δ* caused a low error rate at these positions, showing that the misincorporation by the RT-KTq I614Y polymerase depended on i^6^A37 modification (Fig. 4B).

Surprisingly, we consistently observed a markedly lower error rate in q-treated compared to non-treated wt cells, suggesting reduced i^6^A levels upon q treatment. Therefore, for quantitative assessment, we established a calibration curve by sequencing defined mixtures of tRNA from wt (harboring an unknown absolute level of i^6^A37 in tRNA^Tyr^) with *tit1Δ* (carrying exclusively unmodified A37) under q-free conditions. This enabled us to correlate the error rate at position 37 with i^6^A modification levels relative to wt, which showed a non-linear relationship (Fig. 4C and Supplementary Fig. S7A). The error rate at A37 in wt + q was approx. 22%, which corresponds to a modification level of ∼82.5% compared to cells without q, i.e. a reduction of i^6^A levels by ∼17.5% in the presence of Q34. Of note, analysis of intron-containing tRNA^Tyr^ showed no detectable signal at positions 36 and 37, indicating that i^6^A modification is absent from intron-containing tRNA^Tyr^ (Supplementary Fig. S7B). Altogether, these observations show that Q34 incorporation moderately inhibits i^6^A37 modification of tRNA^Tyr^ in *S. pombe*.

We next asked whether a similar dependence of ms^2^i^6^A levels on Q modification exists for tRNA^Tyr^ in *E. coli*. However, deletion of *tgt* (loss of Q34) or other Q biosynthesis mutants did not alter ms^2^i^6^A37 levels relative to wt (Supplementary Figs S8 and S9). Together with the observation that Q modification levels were unaffected in *∆miaA* cells (Fig. 1D), these results show that Q34 and ms^2^i^6^A37 form independently on tRNA^Tyr^ in *E. coli*.

## Discussion

Queuosine is an evolutionarily conserved tRNA modification that is unusual in eukaryotes, as it depends on the external supply of the queuine nucleobase or the nucleoside from dietary sources. Although widespread across diverse organisms, its loss in both microbes and eukaryotes results in only minor growth defects [23]. Here, we identified a previously unrecognized genetic interaction in *E. coli* between bTGT, which installs Q at position 34, and MiaA, which initiates ms^2^i^6^A formation at position 37. The combined loss of these two modifications caused pronounced growth defects, particularly under thermal stress. These defects were alleviated by overexpression of tRNA^Tyr^, the exclusive substrate for both Q34 and ms^2^i^6^A37, demonstrating that dysfunction of tRNA^Tyr^ underlies the phenotype.

A parallel interaction was observed in *S. pombe* cells lacking Q34 and i^6^A37 modifications, with defects exacerbated by rapamycin. Again, tRNA^Tyr^ overexpression compensated for growth, pointing to a fundamental requirement for this modification pair in maintaining tRNA^Tyr^ function. Furthermore, Q34 modification triggered overexpression of tRNA^Tyr^ in the absence of i^6^A modification in *tit1∆* cells, as well as reduced i^6^A modification levels in tRNA^Tyr^ of wt cells. Altogether, this reveals an unanticipated functional connection between Q34 and i^6^A. This interaction thus joins other tRNA modifications that, when jointly absent, cause growth defects [48–50, 51]. For instance, the absence of m^5^C (*trm4∆*) and N^7^-methyl-guanosine (m^7^G, *trm8∆*) in *Saccharomyces cerevisiae* causes a pronounced growth defect [48]. However, while *trm4∆ trm8∆* result in a rapid decrease of the target tRNA^Val^ through the rapid tRNA decay pathway, we observed here that tRNA^Tyr^ levels in *∆tgt ∆miaA* (*E. coli*) or in *tit1∆* without Q (*S. pombe*) were not reduced. On the contrary, Q modification caused a strong increase in tRNA^Tyr^ levels in *S. pombe tit1∆* cells, but not *∆miaA* bacteria. Of note, aminoacylation levels were unaffected by overexpression or Q and (ms^2^)i^6^A modification both in yeast and bacterial. Thus, the defect reflects the functional consequence of the absence of the two modifications in the anticodon loop, rather than enhanced tRNA decay. Whether the increase results from altered transcription, stability, or turnover remains to be investigated.

On a mechanistic level, our observations indicate that Q34 and ms^2^i^6^A37 act together to ensure decoding accuracy, since the *∆miaA ∆tgt* double mutant exhibited a marked increase in frameshifting at both UAU and UAC tyrosine codons compared to either single mutant. We found no evidence for Q modification of the *virF* mRNA in *S. flexneri* under the conditions investigated here. The combined effect of Q34 and ms^2^i^6^A37 on decoding aligns with reports in *Vibrio cholerae* but is in apparent contrast with a study in *E. coli*, where U-ending codons were most affected by Q deficiency [52, 53]. Methodological divergences may explain the discrepant results: while the prior work measured the collective effect of Q modification on all NAC/U codons, our dual-luciferase system resolves codon-specific contributions. Furthermore, the previous study used a ∆*queF* strain, where Q-tRNAs bear a partially modified preQ_0_ precursor that may itself influence decoding [53]. In contrast, our *∆tgt* strain possesses unmodified G34 at the Wobble position. Summarily, the results show that Q modification influences decoding efficiency in a species- and codon context-dependent manner.

We further observed increased protein aggregation upon combined Q34/ ms^2^i^6^A deficiency in *E. coli*. The altered aggregation pattern reveals a defect in proteostasis that may be driven by increased frameshifting upon combined loss of the two modifications, thus promoting a diminished cellular capacity to respond to external stresses and process misfolded peptides. Whether additional defects of *∆miaA ∆tgt* contribute to the protein aggregation, and whether this effect is seen in eukaryotes, remains to be determined. The increase in protein aggregation is particularly intriguing considering that protein aggregation in neuronal cells causes neurodegenerative disorders such as Alzheimer’s disease [54]. Consistently, female mice lacking the eTGT subunit QTRT1 exhibit defects in learning and memory along with altered hippocampal neuronal composition [20]. Together, these findings suggest that impaired Q and i^6^A modification may contribute to the progression of neurodegenerative disease.

Q34 and (ms^2^)i^6^A37 lie close to each other in the anticodon loop of tRNA^Tyr^ in a geometry reminiscent of Q34 and DNMT2-dependent m^5^C38 in tRNA^Asp^ [14, 17]. Here, we uncovered an unexpected dependence between Q34 and i^6^A37 in *S. pombe* tRNA^Tyr^ in that Q reduced i^6^A37 levels by approximately 17.5% in *S. pombe*, whereas Q did not affect ms^2^i^6^A37 levels in *E. coli* tRNA^Tyr^. Given that eukaryotic cells rely on exogenous sources for Q acquisition, this suggests that environmental Q availability can influence the modification landscape. One possibility is that Q-modified tRNA^Tyr^ is a less efficient substrate for Tit1, which installs i^6^A37. Another possibility is that either modification affects trafficking of the tRNAs between the nucleus and the cytoplasm, thus affecting modification levels, as has been observed in trypanosomes [55]. Also, long-term cultivation of laboratory *S. pombe* strains under Q-free conditions may render sudden Q addition a stress stimulus.

The deep evolutionary conservation of the genetic interaction between Q34 and (ms^2^)i^6^A37 discovered here raises the question of its relevance in higher eukaryotes. Although human cytoplasmic tRNA^Tyr^ carries m^1^G37, its mitochondrial counterpart harbors ms^2^i^6^A37, which is modified by TRIT1 for isopentenylation and CDK5RAP1 for further ms^2^ modification [25]. This architecture resembles the bacterial system from which mitochondria originated, suggesting that a comparable synergy between Q34 and ms^2^i^6^A37 may exist in human mitochondria. If so, Q availability could emerge as a critical determinant of mitochondrial activity, especially in the setting of TRIT1 or CDK5RAP1 deficiencies that impair ms^2^i^6^A synthesis. Pathogenic variants in both genes have been linked to mitochondrial disorders, and the TRIT1–R323Q mutation has been mechanistically connected to defective mitochondrial tRNA modification and translation [30]. Thus, our findings establish an evolutionary framework that advances the molecular understanding of these diseases.

## Supplementary Material

gkag664_Supplemental_File

## Data Availability

High-throughput sequencing data are available in the NCBI GEO database (GSE311025).
