# Supplementary material for: Defective queuosine and i6A/ms2i6A modification of tRNATyr cause frameshifting and protein aggregation
Source: Nucleic Acids Res. 2026 Jul 2;54(12):gkag664. doi: 10.1093/nar/gkag664 (PMC13326639; doi:10.1093/nar/gkag664)
Supplement: gkag664_Supplemental_File [file gkag664_supplemental_file.pdf]

Supplementary Tables

Table S1. ***E. coli*** **strains used in this study**

Strain ID	Genotype	Source
31	*F- Δ(araD-araB)567, ΔlacZ4787 (::rrn B-3), λ–, rph-1, Δ(rhaD-rhaB)568, hsdR5154*	CGSC (BW25113)
26	*F- Δ(araD-araB)567, ΔlacZ4787(::rrnB-3), ΔqueA769::kan, λ-, rph-1, Δ(rhaD-rhaB)568, hsdR514*	CGSC (JW0395-2)
28	*F- Δ(araD-araB)567, ΔlacZ4787(::rrnB-3), λ-, ΔqueF721::kan, rph-1, Δ(rhaD-rhaB)568, hsdR514*	CGSC (JW2765-1)
35	*F- Δ(araD-araB)567, ΔlacZ4787(::rrnB-3), Δtgt::kan, λ-, rph-1, Δ(rhaD-rhaB)568, hsdR514*	this study
40	*F- Δ(araD-araB)567, ΔlacZ4787(::rrnB-3), ΔmiaA::kan, λ-, rph-1, Δ(rhaD-rhaB)568, hsdR514*	this study
43	*F- Δ(araD-araB)567, ΔlacZ4787(::rrnB-3), ΔmiaB::kan, λ-, rph-1, Δ(rhaD-rhaB)568, hsdR514*	this study
42	*F- Δ(araD-araB)567, ΔlacZ4787(::rrnB-3), Δtgt, ΔmiaA::kan, λ-, rph-1, Δ(rhaD-rhaB)568, hsdR514*	this study
58	*F- lambda- ilvG- rfb-50 rph-1 ibpA::ibpA msf-GFP*	this study
116	*F- lambda- ilvG- rfb-50 rph-1 ibpA::ibpA msf-GFP ΔmiaA::kan*	this study
114	*F- lambda- ilvG- rfb-50 rph-1 ibpA::ibpA msf-GFP Δtgt::kan*	this study
119	*F- lambda- ilvG- rfb-50 rph-1 ibpA::ibpA msf-GFP ΔmiaA Δtgt::kan*	this study

Table S2. ***S. flexneri*** **strains used in this study**

Strain ID	Genotype	Source
SF1	WT serotype 5a	(1)
SF3	WT serotype 5a Δ*queC::KanR*	this study
SF2	WT serotype 5a Δ*tgt::KanR*	Lab stock
SF12	WT serotype 5a Δ*queC* Δ*tgt::KanR*	this study

Table S3. ***S. pombe*** **strains used in this study**

Strain ID	Genotype	Source
AEP1	*h- leu1-32 ura4-D18 his3-D3*	Lab stock
AEP618	*h- leu1-32 ura4-D18 his3-D3 tit1Δ::kanMX*	this study
AEP564	*h- leu1-32 ura4-D18 his3-D3 qtr1Δ::NatMX*	this study
AEP619	*h- leu1-32 ura4-D18 his3-D3 qtr1Δ::NatMX tit1Δ::kanMX*	this study

Table S4. Plasmids used in this study

Name	Vector	Description	Source
pAE3087	pKD4	oriRγR6k bla FRT::kan::FRT	Arturo Zychlinsky, MPI for Infection Biology
pAE3750	pKM208	TS AmpR *lacI* Ptac-*red-gam-lacI*	Arturo Zychlinsky, MPI for Infection Biology
pAE3777	pCP20	FLP recombinase	Marc Erhardt, Humboldt-Universität zu Berlin
pAE3779	pTrc99a-FFA	promoter p*Trc*	Marc Erhardt, Humboldt-Universität zu Berlin
pAE3778	pBAD33.1	promoter p*BAD*	Marc Erhardt, Humboldt-Universität zu Berlin
pAE3781	pTrc99a-FFA	pTrc-*shigella* tRNATyr	This study
pAE3819	pTrc99a-FFA	pTrc-*E. coli miaA*	This study
pAE3290	pUC18	*Shigella flexneri* 5a (M90T) tgt-1xHA	This study
pAE3788		Rluc- in frameshifting TAT -Fluc	Juan Alfonzo (Brown University) (2)
pAE3787		Rluc- in frameshifting TAC -Fluc	Juan Alfonzo (Brown University)
pAE3786		Rluc- +1frameshifting TyrTAT -Fluc	Juan Alfonzo (Brown University)
pAE3785		Rluc- +1frameshifting TyrTAC -Fluc	Juan Alfonzo (Brown University)
pAE3823	pBAD33.1	Rluc- +1frameshifting CCC-TAT -Fluc	This study
pAE3822	pBAD33.1	Rluc- +1frameshifting CCC-TAC -Fluc	This study
pAE3826	pBAD33.1	Rluc- in frameshifting CCC-TAT -Fluc	This study
pAE3825	pBAD33.1	Rluc- in frameshifting CCC-TAC -Fluc	This study
pAE3861	pREP3x	*S. pombe* $tRNA^{Tyr}$ with 300 bp upstream and downstream	This study
RAC310	pRACmsfGFP	oriRγR6k bla FRT::msfGFP-kan::FRT	This study

Table S5. Oligonucleotides used in this study

Primer ID	Name	Sequence 5' to 3'
4614	queC_con_145.fwd	TGGCGCTGAAACTGGGAGC
4615	queC_con_789.rev	GAAGGGGAATTGCGCGAAA
4616	queC_KO_shigella2.fw	CAGGATTATCTATGAAACGTGCTGTCGTTGTGTTCAGTGGAGGCCAGGATCGATTGTGTAGGCTGGAGCTGCTTC
4617	queC_KO_shigella2.re	ACACCCGGAATAATTACCTCAACCCGGTTTTCTGCTTCATCGCTGCCATCACATGGGAATTAGCCATGGTCCATA

4620	E.coli_tgt_knockout	CGCTGGTTTAAAACGTTGGACTGTTTTTCTGACGTAGTGGAGAAAAAATGCGATTGTGTAGGCTGGAGCTGCTTC
4621	E.coli_tgt_knockout	AGCTCATTAAATTTCCCTCATTATTAATATTAATCAACGTTCAAAGGTGGATGGGAATTAGCCATGGTCCATA
4622	E.coli_tgt_100.fwd	ATCACGTACAATCCGCAGG
4623	E.coli_tgt_1225.rev	CAAAATCAAAGACATCGGGC
3368	Neo/Kan_fwd.	CGTTGGCTACCCGTGATATT
3369	Neo/Kan_rev.	GCCCAGTCATAGCCGAATAG
4643	MiaA_KO_sf.fwd	TTACAAAGGGATGGATATCGGGACGGCGAAGCCGAACGCTGAAGAGTTACCGATTGTGTAGGCTGGAGCTGCTTC
4644	MiaA_KO_sf.rev	CGTATGAGATTTCGCCTTCAAGGTAAGACCACATCTGGCGATAACCCACGCAATGGGAATTAGCCATGGTCCATA
4645	MiaA_-37_sf.fwd	TCTGTTGATTTACATCCGGC
4646	MiaA_990_sf.rev	CTGAAAAAATTGCGCACGATACG
4647	MiaB_KO_sf.fwd	GAACACCTGCTCAATCCGCGAGAAGGCTCAGGAAAAAGTCTTCCATCAGTCGATTGTGTAGGCTGGAGCTGCTTC
4648	MiaB_KO_sf.rev	GAATTTACCGATCATATCCGGTGTGCCCTCGAAGTTGACCACGCGGTTATTTATGGGAATTAGCCATGGTCCATA
4649	MiaB_-37_sf.fwd	CCTGCATTCCTGGCTACTAT
4650	MiaB_1466_sf.rev	GAGAGAAAAAACAAGGCCCAC
4815	Tit1_1342_fwd	GGGCTAAGCTTATAAATACCCG
4816	Tit1_1220_rev	CATGATTAAAAGCACCACAGAG
4824	tit1 UP FP	CAG TTG TAT CAA TAC CAG CCT G
4825	tit1 UP RP	GGG TAT TCT GGG CCT CCA TGT CCG AAT CAA GCT TAG ACT AAC AAA TCC
4826	tit1 KanMX FP	GGA TTT GTT AGT CTA AGC TTG ATT CGG ACA TGG AGG CCC AGA ATA CCC
4827	tit1 KanMX RP	CTT CAA TAA TGA CCA TTC TCC ATA GCC AGT ATA GCG ACC AGC ATT CAC
4828	tit1 DN FP	GTG AAT GCT GGT CGC TAT ACT GGC TAT GGA GAA TGG TCA TTA TTG AAG
4829	tit1 DN RP	CCT TTG GCA ATT GAC CTA CAA G
RAC492	ibpAmsfGFP_VFTF	cgcgtgattccggaagcgaaaaaaccgcgccgtatcgaaatcaacGGTGGCAGCGGTATGAGCAAAG
RAC493	ibpAmsfGFP_RVTV	cggcgagcatggagatgtcaggccgcgccaggcggccttagggaacatatgaatatcctccttagttcc

Table S6. Probes used for Northern blotting

Primer ID	Name	Sequence 5' to 3'
3997	Biot_tRNAAsp_pombe	biotin-GGGCTGCAAGCGTGACAGG

4651	Biotin_Tyr_Shigella	Biotin-CGGCAGATTTACAGTCTGCTCCC
4949	Sp_gentRNATyr_biotin	biotin-GCGACCAACCGGTTTACAGCCG

Table S7. Oligonucleotides used for ms^2i^6A detection

Primer ID	Name	Sequence 5' to 3'
3843	S.f._RT_tRNATyr	CTCAACTGGATTGGCTNNNNNGATAA ATCCAGTTGAGTGGTGGTGGTGG
3851	S.f._tRNATyr_fwd	GGTGGGGTTCCCGAG
3858	Stemloop Index N_rev	CGATCAnnnnCTCAACTGGATTGGCT

Note: Nucleotides marked as ‘n’ are the barcode region.

Table S8. Oligonucleotides used for i^6A detection

Primer ID	Name	Sequence 5' to 3'
3838	RT-Primer_tRNATyr	CTCAACTGGATTGGCTNNNNNGATA AATCCAGTTGAGTGGTCTCCTGAG
3846	tRNATyr_fwd	CTCCTGATGGTGTAGTTG
3858	Stemloop Index N_rev	CGATCAnnnnCTCAACTGGATTGGCT

Table S9. Oligonucleotides used for *virF* RT-qPCR

Primer ID	Name	Sequence 5' to 3'
4632	virF_qPCR.fwd	CAG CTG TTT CTG ATG AGG AAG
4633	virF_qPCR.rev	CTC CAA TCG TTT TCT AAC AGC
3840	RT-Primer_virF	CTCAACTGGATTGGCTNNNNNGATA AATCCAGTTGAGTGGTTAAAATTT
3841	S.f._RT_tRNAAsp	TCAACTGGATTGGCTNNNNNGATAAA TCCAGTTGAGTGGTGGCGGAAC
3849	S.f._tRNAAsp_fwd	GGAGCGGTAGTTCAGTC
3858	Stemloop Index N_rev	CGATCAnnnnCTCAACTGGATTGGCT

Table S10. SRM table for nucleoside analysis

Compound	Retention Time (min)	RT Window (min)	Precursor (m/z)	Product (m/z)	Collision Energy (V)	RF Lens (V)
C	2	4	244	112	11	42
U	2	4	245	113	10	30
Y	2	4	245	209	10	30
D	2	4	247	115	10	30
cmo^5U	2.68	4	319	187	10	58
acp^3U	2.95	4	346	214	10	58
nm^5U	3	6	274	142	35	96

Fapy-A	3	6	286	154	15	54
Gh	3	6	290	158	15	54
Fapy-G	3	6	302	170	15	54
cmnm^{5}U	3	6	332	200	10	58
mnm^{5}U	3	4	288	156	10	30
dC	4	8	228	112	14	54
s^{2}C	4	8	260	128	11	42
m^{1}A	4.37	6	282	150	20	68
cmnm^{5}s^{2}U	5	10	348	216	10	58
mnm^{5}s^{2}U	5	4	304	172	10	51
m^{5}C	6	4	258	126	14	46
Cm	7	4	258	112	12	41
m^{7}G	7.5	4	298	166	16	50
**Compound**	**Retention Time (min)**	**RT Window (min)**	**Precursor (m/z)**	**Product (m/z)**	**Collision Energy (V)**	**RF Lens (V)**
cmnm^{5}Um	8	6	346	200	10	58
I	8.3	4	269	137	10	40
m^{5}U	8.69	4	259	127	10	30
8-oxo-G	10	20	300	168	16	49
s^{4}U	10.78	6	261	129	10	58
G	12	10	284.2	152	14	46
dG	13	15	268	136	17	63
Um	13	6	259	113	10	30
m^{3}U	13	6	259	127	10	30
oQ	15.21	12	426	163	29	84
oQ1	15.21	12	426	295	16	49
m^{4}Cm	15.35	6	272	126	11	46
Q	17.6	14	410	163	31	96
Q1	17.6	14	410	295	16	49
dT	18	20	243	127	14	54
k^{2}C	18	8	372	240	12	41
m^{1}G	18.3	8	298	166	16	50
Gm	19.27	6	298	152	11	50
m^{2}G	20.4	12	298	166	16	50
ac^{4}C	20.5	12	286	154	10	46
preQ0	23	46	308	176	31	96
preQ1	23	46	312	180	31	96

A	25	20	268	136	17	63
8oxo-A	25	14	284	152	15	54
dA	28	20	252	136	14	54
t^6A/d-allo	29.3	18	413	281	31	96
$m^{2,2}G$	29.5	16	312	180	15	60
Am	29.6	12	282	136	17	63
m^2A	29.7	8	282	150	20	74
m^6A	30.3	8	282	150	20	74
m^6t^6A	32.3	16	427	295	31	96
ms^2A	33	14	314	182	31	96
ct^6A	33	6	395	263	31	96
$m^{6,6}A$	33.4	6	296	164	28	79
io^6A	33.55	4	352	220	31	96
ms^2io^6A	36.3	4	398	266	31	96
i^6A	36.5	6	336	204	31	96
ms^2i^6A	40.9	6	382	250	31	96

Supplementary Figures

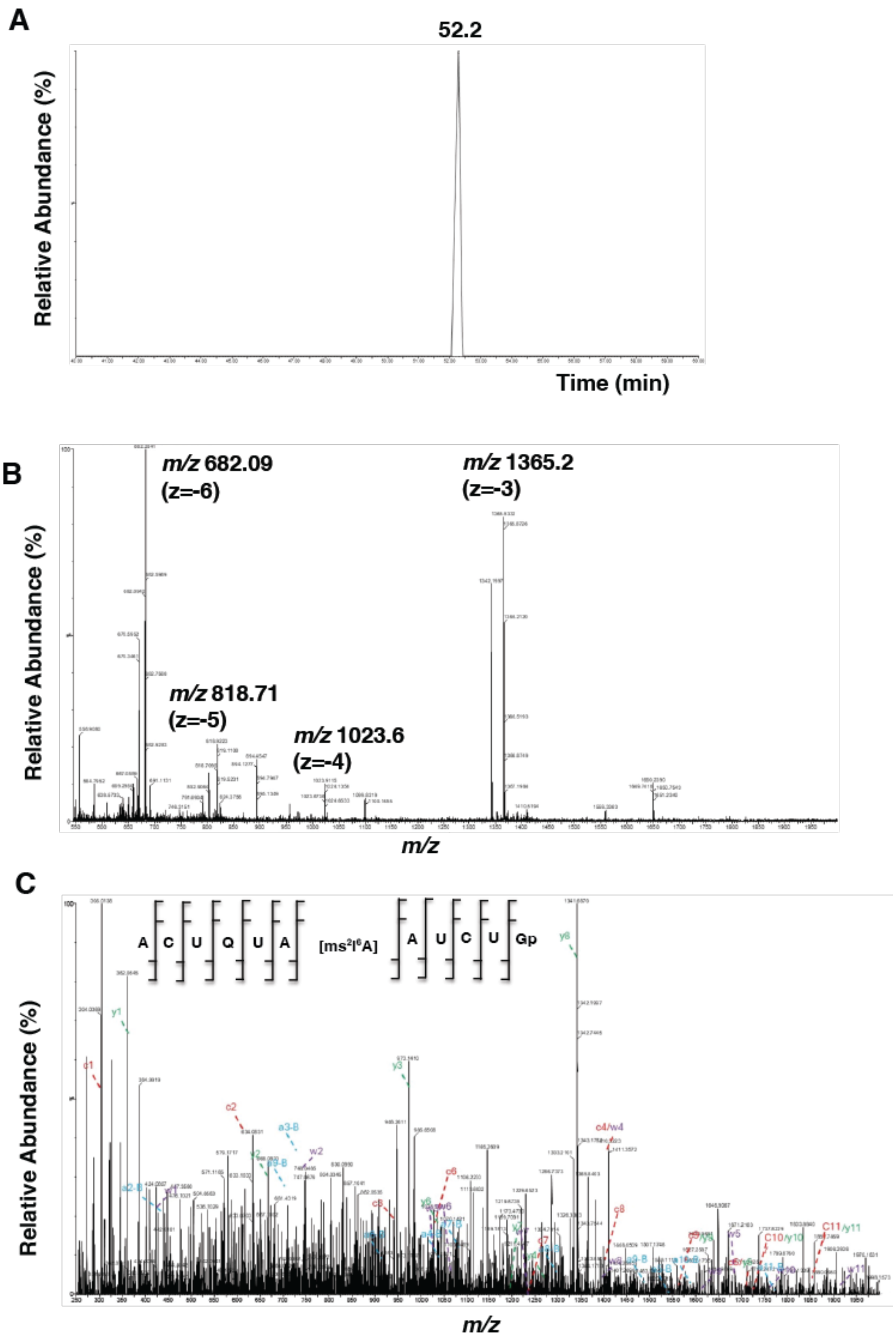

Fig. S1. Co-occurrence of Q34 and ms^2i^6A37 modification on *E. coli* $tRNA^{Tyr}$ as determined by LC-MS/MS analysis of RNase T1-digested *E. coli* tRNAs. (*A*) Extracted ion chromatogram (XIC) of the fragment ACUQUA[ms^2i^6A]AUCUGp derived from $tRNA^{Tyr}$. (*B*) Mass spectrum corresponding to XIC at 52.2 min. (*C*) MS/MS of m/z 1365.2 with sequence informative fragment ions labeled.

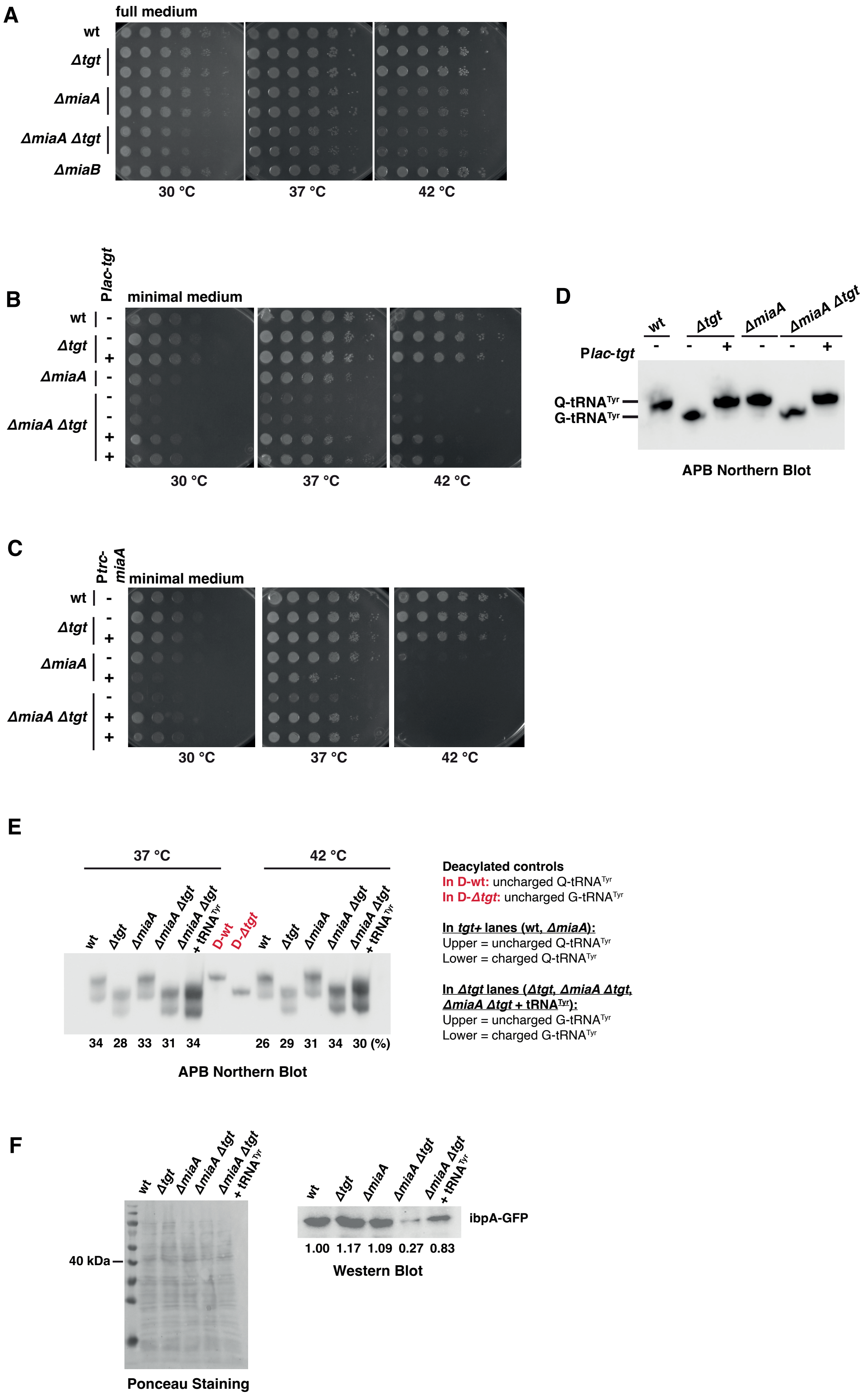
A
full medium
wt
Δtgt
ΔmiaA
ΔmiaA Δtgt
ΔmiaB
30 °C
37 °C
42 °C
B
Plac-tgt
minimal medium
wt
Δtgt
ΔmiaA
ΔmiaA Δtgt
30 °C
37 °C
42 °C
C
Ptrc-miaA
minimal medium
wt
Δtgt
ΔmiaA
ΔmiaA Δtgt
30 °C
37 °C
42 °C
D
wt
Δtgt
ΔmiaA
ΔmiaA Δtgt
Plac-tgt
Q-tRNATyr
G-tRNATyr
APB Northern Blot
E
37 °C
42 °C
wt
Δtgt
ΔmiaA
ΔmiaA Δtgt
ΔmiaA Δtgt + tRNATyr
D-wt
D-Δtgt
34 28 33 31 34
26 29 31 34 30 (%)
APB Northern Blot
Deacylated controls
In D-wt: uncharged Q-tRNATyr
In D-Δtgt: uncharged G-tRNATyr
In tgt+ lanes (wt, ΔmiaA):
Upper = uncharged Q-tRNATyr
Lower = charged Q-tRNATyr
In Δtgt lanes (Δtgt, ΔmiaA Δtgt, ΔmiaA Δtgt + tRNATyr):
Upper = uncharged G-tRNATyr
Lower = charged G-tRNATyr
F
wt
Δtgt
ΔmiaA
ΔmiaA Δtgt
ΔmiaA Δtgt + tRNATyr
40 kDa
Ponceau Staining
ibpA-GFP
1.00 1.17 1.09 0.27 0.83
Western Blot

Fig. S2. Complementation with *tgt* or *miaA* rescues the growth defect of Δ*miaA* Δ*tgt*. (*A*) Growth assay of *E. coli* strains lacking the tRNA modification genes *tgt* and *miaA* on LB plates. The indicated *E. coli* strains were serially 10-fold diluted and spotted on LB plates, and incubated at 30°C, 37°C, or 42°C for 12 h. (*B*) Rescue of growth defects of Δ*miaA* Δ*tgt* by complementation with *tgt* under the control of the P*lac* promoter. Strains were transformed with a plasmid expressing the corresponding gene (+) or with an empty vector (-) as a control. Transformants were serially 10-fold diluted and spotted onto M9 plates supplemented with vitamins, trace elements, ampicillin, and 0.5 mM IPTG to induce *tgt* expression. The strains were incubated at 30°C, 37°C, or 42°C for 18 h. (*C*) Rescue of growth defects in the Δ*miaA* Δ*tgt* mutant by complementation with *miaA* using P*trc* promoter. The Δ*miaA* Δ*tgt* mutant carrying plasmid-derived *miaA* exhibited improved growth at 30°C and 37°C. Of note, introduction of *miaA* reduced the growth of Δ*miaA*, which is consistent with previous reports that excessive *miaA* expression is detrimental. The results here were obtained in the absence of IPTG, which is used to induce the P*trc* promoter, which may suggest that the basal activity of the P*trc* promoter drives *miaA* expression above native levels. (*D*) APB Northern blot analysis of $tRNA^{Tyr}$ was performed to confirm that the plasmid-derived *tgt* gives rise to a functional bTGT capable of Q formation. For this assay, transformants were cultivated in M9 medium supplemented with vitamins, trace elements, ampicillin at 37ºC, and 0.5 mM IPTG to induce *tgt* expression. Small RNAs were extracted and analyzed by APB Northern blot using a probe specific for $tRNA^{Tyr}$. The migration of Q-modified tRNAs in APB gels is slower than that of G-containing tRNAs. (*E*) Aminoacylation of $tRNA^{Tyr}$ was unaffected in *E. coli* strains lacking Q34 and/or ms^2i^6A37. The numbers below the lanes indicate the percentage of charged $tRNA^{Tyr}$, calculated as the charged signal divided by the sum of charged and uncharged signals. Δ*miaA* Δ*tgt* + $tRNA^{Tyr}$ indicates Δ*miaA* Δ*tgt* strain overexpressing $tRNA^{Tyr}$. D-wt indicates the deacylated wt sample. D-Δ*tgt* indicates the deacylated Δ*tgt* sample. (*F*) Analysis of total IbpA-GFP levels. IbpA-GFP protein abundance was analyzed by anti-GFP Western blot in the indicated *E. coli* strains. Ponceau staining was used as a loading control. Values below the blot indicate IbpA-GFP signal normalized to Ponceau staining and relative to wt.

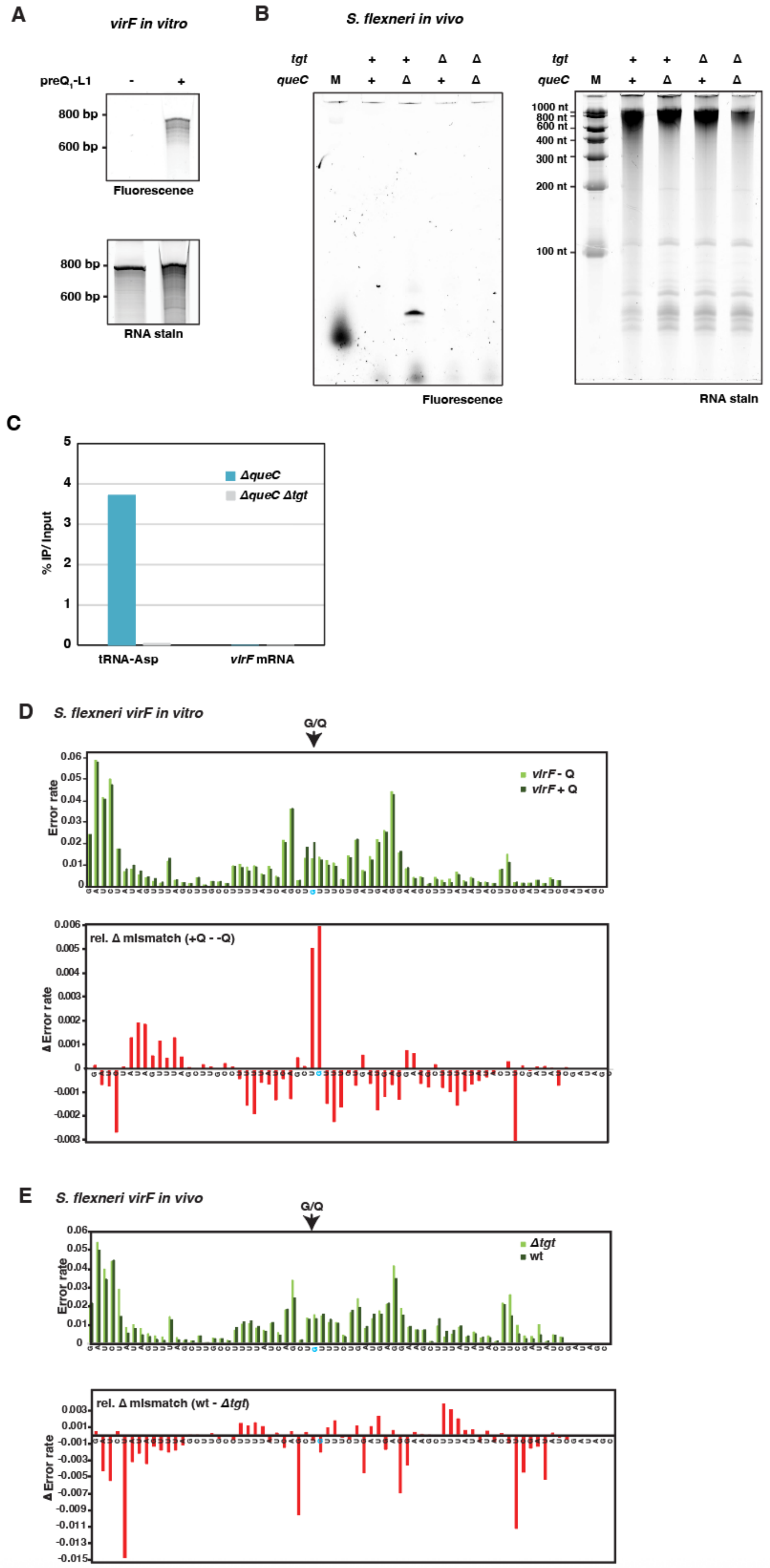
A
virF in vitro
preQ1-L1
-
+
800 bp
600 bp
Fluorescence
800 bp
600 bp
RNA stain
B
S. flexneri in vivo
tgt
queC
M
Fluorescence
1000 nt
800 nt
600 nt
400 nt
300 nt
200 nt
100 nt
RNA stain
C
% IP/ Input
ΔqueC
ΔqueC Δtgt
tRNA-Asp
virF mRNA
D
S. flexneri virF in vitro
G/Q
Error rate
virF - Q
virF + Q
rel. Δ mismatch (+Q - -Q)
Δ Error rate
E
S. flexneri virF in vivo
G/Q
Error rate
Δtgt
wt
rel. Δ mismatch (wt - Δtgt)
Δ Error rate

Fig. S3. Q modification was not detectable in *S. flexneri virF* mRNA *in vivo*. (*A*) *In vitro*-transcribed *virF* RNA is modified with preQ$_1$-L1 by human TGT. *virF* RNA was incubated in the absence (-) or presence (+) of preQ$_1$-L1 and human TGT, and the incorporation of preQ$_1$-L1 was detected via copper(I)-catalyzed azide alkyne cycloaddition (CuAAC) using AlexaFluor594-alkyne. RNAs were resolved on 10% polyacrylamide/8M urea denaturing gels. Top: fluorescence detection of AlexaFluor594 at 532 nm. Bottom: total RNA staining as a loading control. (*B*) preQ$_1$-L1 incorporation into *S. flexneri* tRNAs *in vivo*. All strains (wt, Δ*queC*, Δ*tgt*, and Δ*queC* Δ*tgt*) were cultured in M9 medium supplemented with preQ$_1$-L1 (10 µM). Total RNA was extracted, labeled with AlexaFluor594, and analyzed by fluorescence gel electrophoresis as described in (*A*). M: molecular weight marker. (*C*) *S. flexneri virF* is not modified with preQ$_1$-L1 *in vivo*. Total RNA from Δ*queC* and Δ*queC* Δ*tgt* cultured with preQ$_1$-L1 was subjected to biotin-click followed by enrichment using streptavidin-coated magnetic beads (IP). Enriched RNA was used for RT-qPCR analysis of *virF* mRNA and $tRNA^{Asp}$ (as a positive control), with input normalization using metabolically labeled, biotin-clicked RNA without immunoprecipitation. $tRNA^{Asp}$ was markedly enriched in the IP fraction compared with the input sample in the Δ*queC* strain, but not in Δ*queC* Δ*tgt*. No enrichment of *virF* mRNA was detected either in Δ*queC* or Δ*queC* Δ*tgt.* (*D*) and (*E*) Reverse transcription using the KTq I614Y leads to an increased error rate in *in vitro*-transcribed and Q-modified *virF* mRNA, but not in *virF* mRNA *ex cellulo*. Reverse transcription was performed on *in vitro*-transcribed and Q-modified *virF* mRNA and *virF* mRNA from total RNA of wt and Δ*tgt* cultured in rich medium. Resulting cDNAs were amplified and sequenced. Comparison of the error rates of unmodified and Q-modified RNA. (*D*) *in vitro* and (*E*) *virF* mRNA *ex cellulo* (top plots). The difference in error rates in the presence and absence of Q is given (bottom plots, red bars).

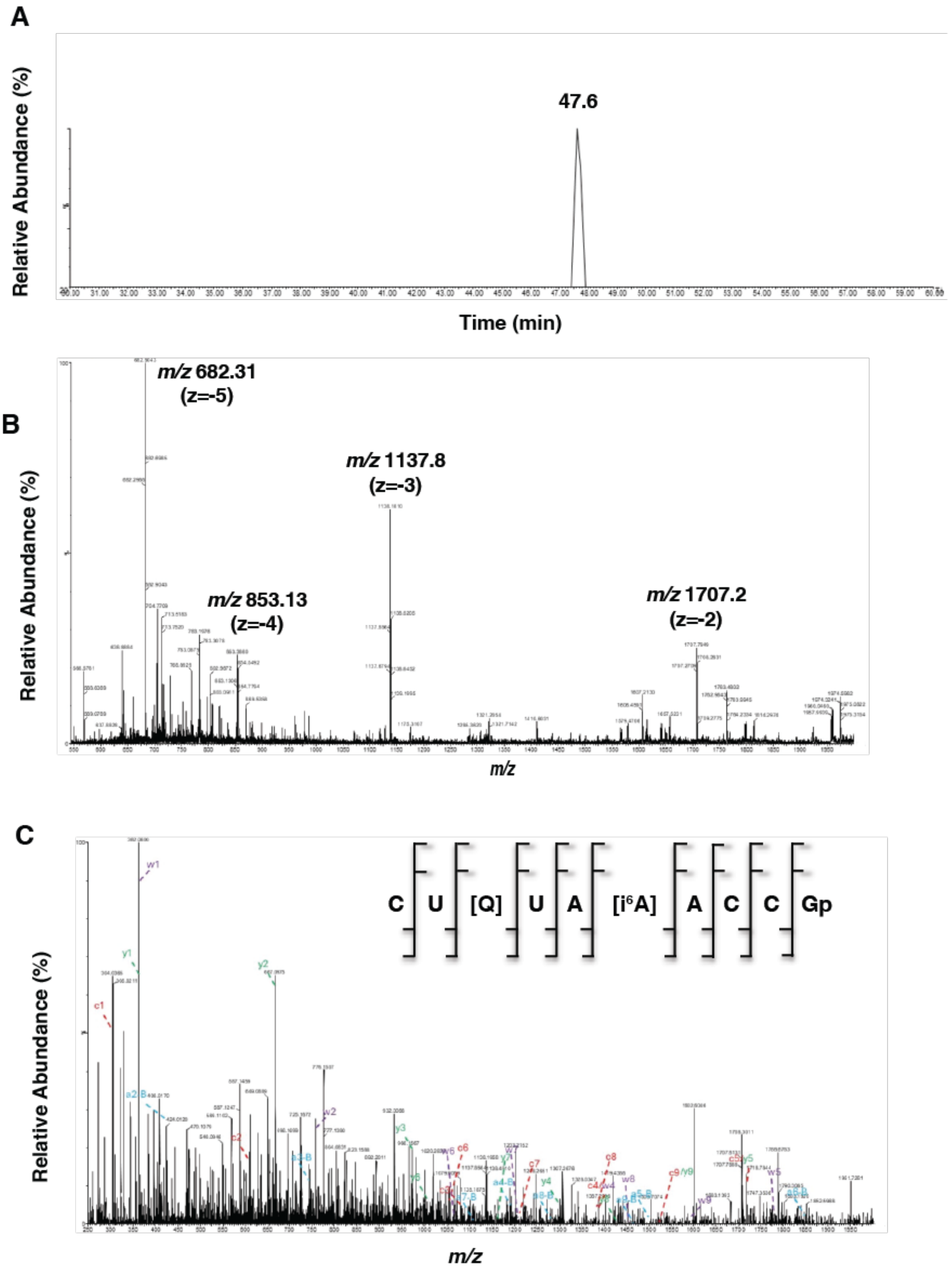

Fig. S4. Co-occurrence of Q34 and i[6]A37 modification on *S. pombe* tRNA[Tyr] by LC-MS/MS analysis of RNase T1-digested *S. pombe* tRNAs. (*A*) Extracted ion chromatogram (XIC) of the fragment CUQUA[i[6]A]ACCGp derived from tRNA[Tyr]. (*B*) Mass spectrum corresponding to XIC at 47.6 min. (*C*) MS/MS of m/z 682.31 with sequence informative fragment ions labeled.

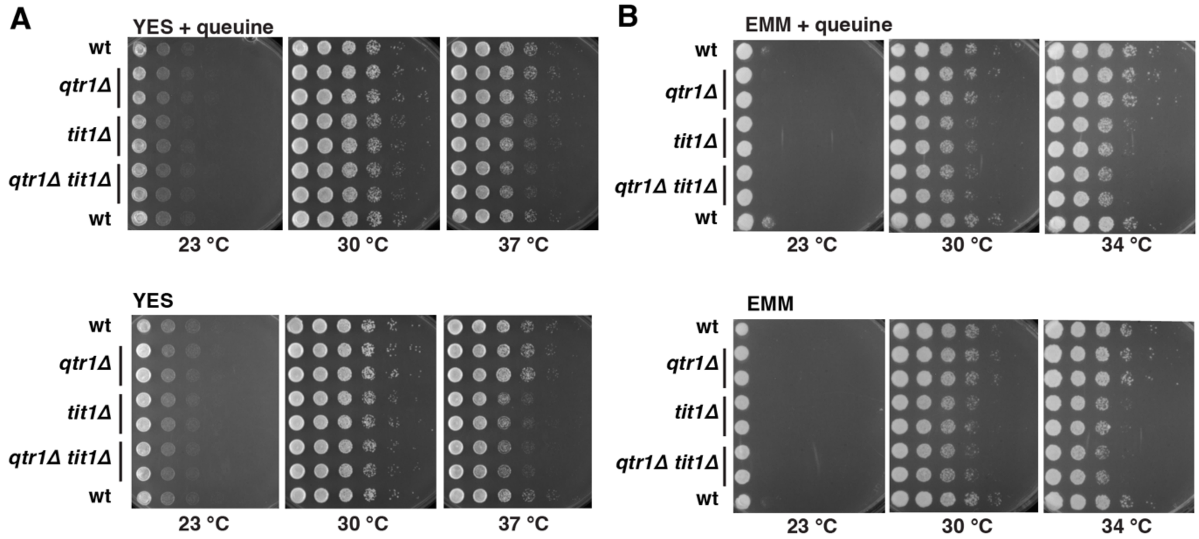

Fig. S5. Absence of Q34 modification causes a mildly enhanced growth defect in *tit1*∆ *S. pombe* strains, which lack i^{6}A, on rich (YES) and minimal medium (EMM). Serial 6-fold dilutions of the indicated *S. pombe* strains were spotted onto YES plates (*A*) or EMM plates (*B*) with (top) or without (bottom) q supplementation (100 nM). Plates were incubated at the indicated temperatures.

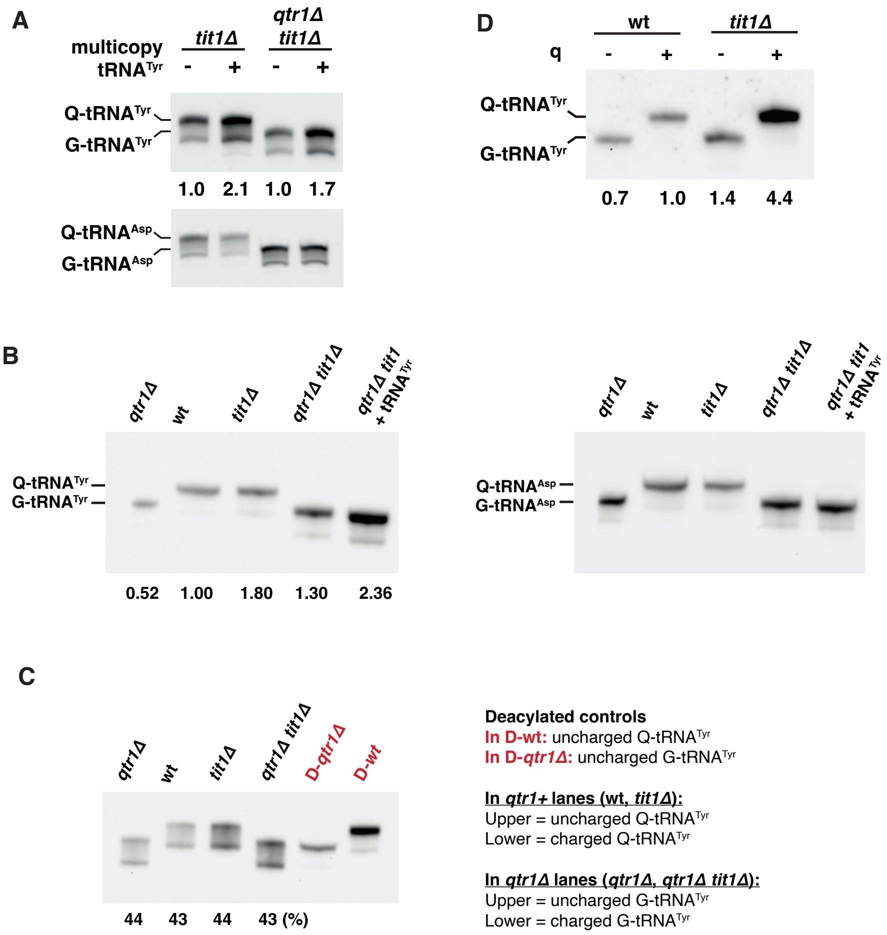

Fig. S6. Q34 modification causes overexpression of tRNA^{Tyr} in *S. pombe tit1*Δ. *(A)* Expression of tRNA^{Tyr} from a multicopy plasmid causes 2.1 and 1.7-fold overexpression. Cells were grown at 30ºC in the presence of q, and small RNAs were analyzed by APB Northern blot using a probe for tRNA^{Tyr} (top) and for tRNA^{Asp} (bottom) as a control. Values below the lanes show the expression of tRNA^{Tyr} from the multicopy plasmid relative to the vector control. *(B)* The levels of tRNA^{Tyr} levels were not reduced in *S. pombe qtr1*Δ *tit1*Δ at elevated temperatures. Northern blot analysis was performed to assess the levels of tRNA^{Tyr} in the indicated *S. pombe* strains cultivated in EMM medium with rapamycin and q at 34°C, using tRNA^{Asp} as a loading control. Q- and G-containing tRNA species are indicated on the left. The values below the tRNA^{Tyr} blot indicate the quantified tRNA signal normalized to wt. *(C)* tRNA^{Tyr} aminoacylation levels in *S. pombe* are unaffected by Q or i^{6}A modification. S. pombe strains were cultured in YES medium with q at 34°C. tRNA^{Tyr} charging was analyzed in the indicated strains. D-wt and D-*qtr1*Δ indicate deacylated wt and *qtr1*Δ samples, respectively, and serve as migration controls for uncharged Q-tRNA^{Tyr} and uncharged G-tRNA^{Tyr}. *(D)* Q34 modification causes increased expression of tRNA^{Tyr} in *tit1*Δ. Strains were grown in the absence (-) or presence (+) of q at 30ºC, and small RNAs were analyzed by APB Northern blot using a probe for tRNA^{Tyr}. Values below each lane represent the expression level of tRNA^{Tyr} relative to wt cultured with q (set as 1).

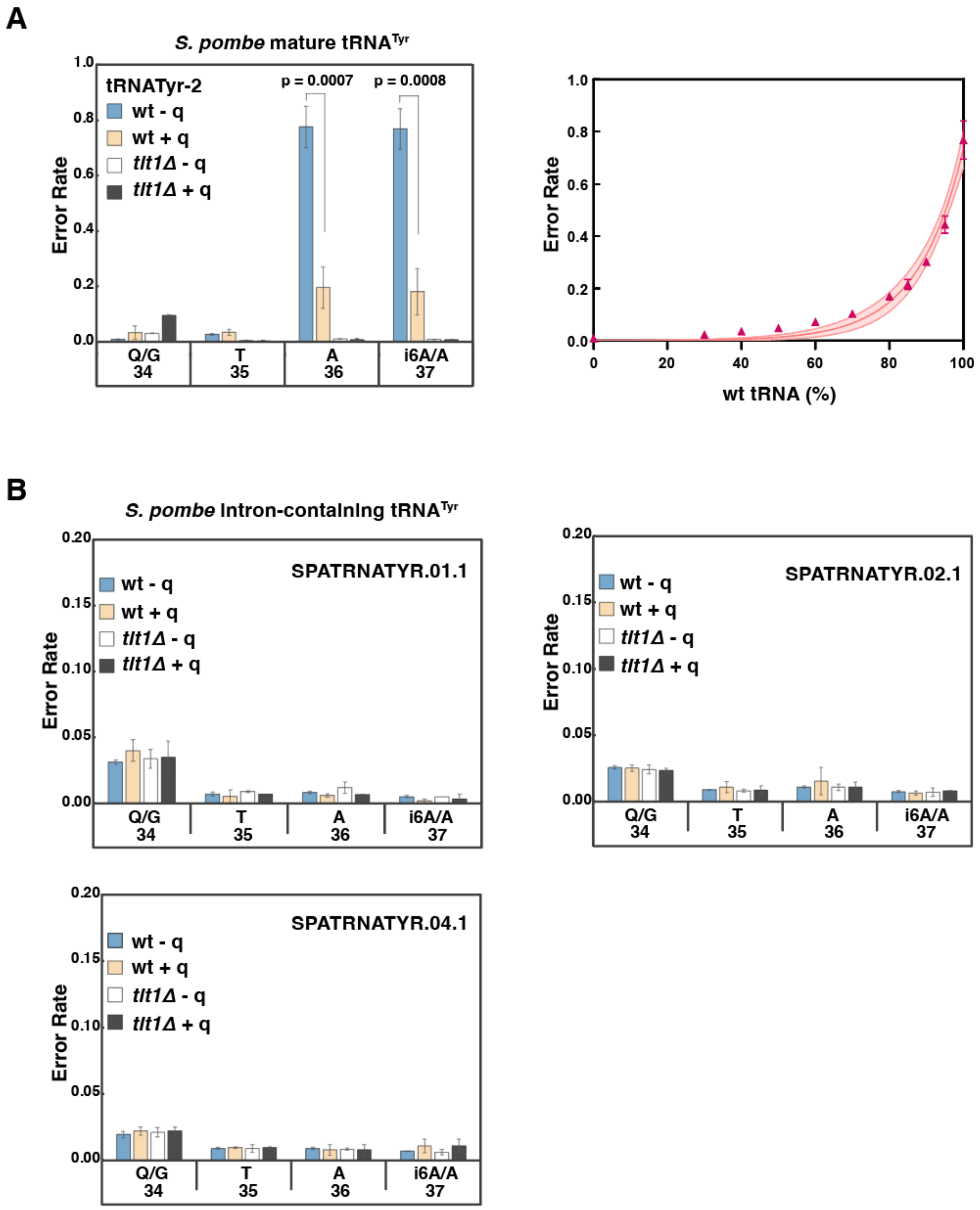

Fig. S7. Detection of i^{6}A37 modification in *S. pombe* tRNA^{Tyr}. (*A*) Q modification reduces the i^{6}A37 level in the tRNATyr-2 isodecoder (see Figure 4 for the isodecoder tRNATyr-1). Left: Error profiles of tRNATyr-2 were generated from iodine-treated small RNA samples, as described in Figure 4. wt-q: wt cultivated in YES without q supplementation; wt+q: wt cultivated in YES with q; *tit1Δ*-q: *tit1Δ* cultivated in YES without q; *tit1Δ*+q: *tit1Δ* cultivated in YES with q. Data represent the mean ± SD of three biological replicates per sample. Right: Calibration curves were generated by mixing iodine-treated RNA from wt-q and *tit1Δ*-q samples at defined ratios and used to establish a relatively quantitative relationship between error rate at position 37 and i^{6}A levels relative to that of wt-q. The red curve in the panel represents an exponential fit (R^2 = 0.9787 for the red curve), with shaded areas indicating the 95% confidence intervals. (*B*) i^{6}A is absent in *S. pombe* intron-containing tRNA^{Tyr}. The three tRNA^{Tyr} species analyzed represent all three intron-containing tRNA^{Tyr} genes in *S. pombe*.

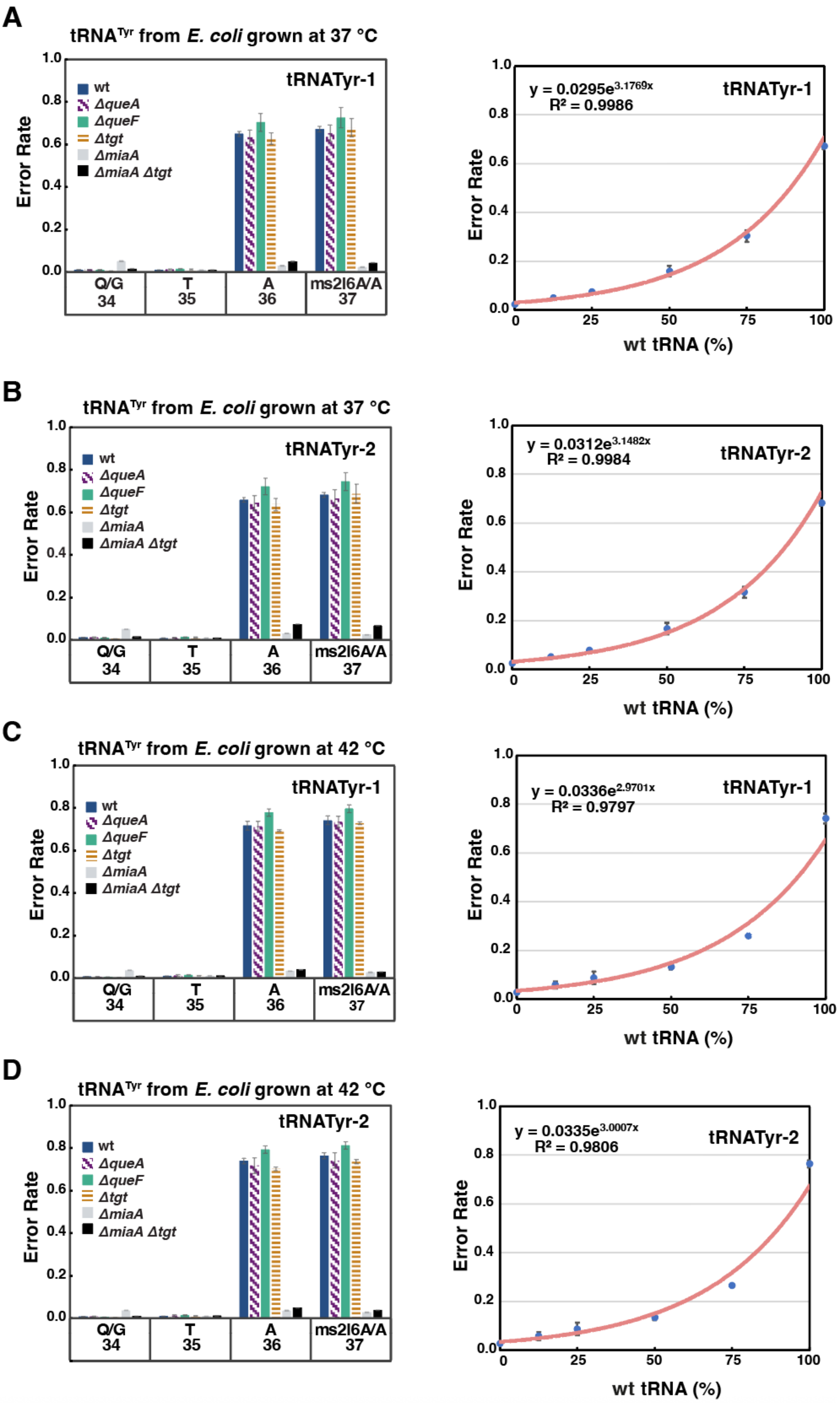
A
tRNATyr from E. coli grown at 37 °C
tRNATyr-1
Error Rate
1.0
0.8
0.6
0.4
0.2
0.0
wt
ΔqueA
ΔqueF
Δtgt
ΔmiaA
ΔmiaA Δtgt
Q/G 34
T 35
A 36
ms2I6A/A 37
y = 0.0295e3.1769x
R² = 0.9986
wt tRNA (%)
0
25
50
75
100
B
tRNATyr from E. coli grown at 37 °C
tRNATyr-2
y = 0.0312e3.1482x
R² = 0.9984
C
tRNATyr from E. coli grown at 42 °C
tRNATyr-1
y = 0.0336e2.9701x
R² = 0.9797
D
tRNATyr from E. coli grown at 42 °C
tRNATyr-2
y = 0.0335e3.0007x
R² = 0.9806

Fig. S8. ms^2i^6A37 levels in *E. coli* $tRNA^{Tyr}$ are unaffected by the modification state at position 34 (unmodified, Q precursors or Q). Error rates attributable to ms^2i^6A remain consistent between wt and Q-biosynthesis pathway mutants in both $tRNA^{Tyr}$ isodecoders, regardless of cultivation at 37°C (*A* and *B*) or 42°C (*C* and *D*). In *E. coli*, the $tRNA^{Tyr}$ position 34 contains Q in the wt strain, $preQ_1$ in the *ΔqueA*, $preQ_0$ in the *ΔqueF*, and G in the Δ*tgt* strain. (*A*), (*B*), (*C*) and (*D*) Left: Error rate profiles of $tRNA^{Tyr}$. Error rate profiles were obtained from small RNA samples reverse transcribed with engineered RT-KTq I614Y in the presence of reduced dTTP to enhance misincorporation, followed by PCR and high-throughput sequencing. Data represent the mean ± SD of three biological replicates per sample. Right: Exponential fit (red curve) between the error rate at position 37 and the proportion of wt-derived small RNAs in the calibration mixtures. Calibration samples, generated by mixing RNA from wt and Δ*miaA* at the indicated ratios, were used to establish a quantitative relationship between error rate and relative ms^2i^6A levels (normalized to that in wt).

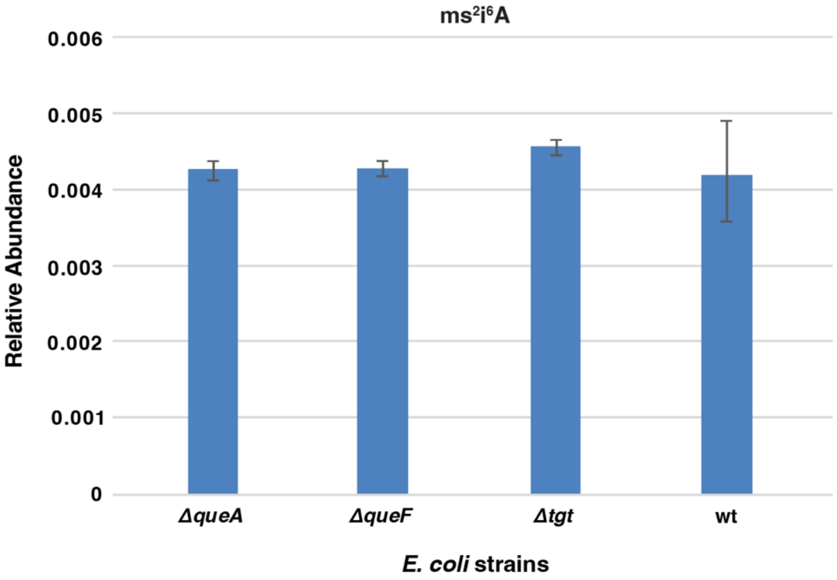

Fig. S9. LC-MS/MS nucleoside analysis of ms^2i^6A levels in *E. coli* wt and strains with defects in Q biosynthesis. Deletion of *tgt* (loss of Q34) or other Q biosynthesis genes in *E. coli* did not affect the overall cellular levels of ms^2i^6A compared with wt (n = 3 biological replicates, bars give standard error of the mean).

References

1. Sansonetti, P.J., Kopecko, D.J. and Formal, S.B. (1982) Involvement of a plasmid in the invasive ability of Shigella flexneri. *Infect Immun*, **35**, 852-860.
2. Dixit, S., Kessler, A.C., Henderson, J., Pan, X., Zhao, R., D'Almeida, G.S., Kulkarni, S., Rubio, M.A.T., Hegedusova, E., Ross, R.L. *et al.* (2021) Dynamic queuosine changes in tRNA couple nutrient levels to codon choice in Trypanosoma brucei. *Nucleic Acids Res*, **49**, 12986-12999.
